# Maternal Liver Metabolic Response to Chronic Vitamin D Deficiency Is Determined by Mouse Strain Genetic Background

**DOI:** 10.1093/cdn/nzaa106

**Published:** 2020-06-20

**Authors:** Jing Xue, Elizabeth K Hutchins, Marwa Elnagheeb, Yi Li, William Valdar, Susan McRitchie, Susan Sumner, Folami Y Ideraabdullah

**Affiliations:** Department of Genetics, School of Medicine, University of North Carolina at Chapel Hill, Chapel Hill, NC, USA; Nutrition Research Institute, University of North Carolina at Chapel Hill, Kannapolis, NC, USA; Department of Nutrition, Gillings School of Public Health, University of North Carolina at Chapel Hill, Chapel Hill, NC, USA; Department of Genetics, School of Medicine, University of North Carolina at Chapel Hill, Chapel Hill, NC, USA; Nutrition Research Institute, University of North Carolina at Chapel Hill, Kannapolis, NC, USA; Department of Genetics, School of Medicine, University of North Carolina at Chapel Hill, Chapel Hill, NC, USA; Department of Genetics, School of Medicine, University of North Carolina at Chapel Hill, Chapel Hill, NC, USA; Lineberger Comprehensive Cancer Center, University of North Carolina at Chapel Hill, Chapel Hill, NC, USA; Nutrition Research Institute, University of North Carolina at Chapel Hill, Kannapolis, NC, USA; Department of Nutrition, Gillings School of Public Health, University of North Carolina at Chapel Hill, Chapel Hill, NC, USA; Nutrition Research Institute, University of North Carolina at Chapel Hill, Kannapolis, NC, USA; Department of Nutrition, Gillings School of Public Health, University of North Carolina at Chapel Hill, Chapel Hill, NC, USA; Department of Genetics, School of Medicine, University of North Carolina at Chapel Hill, Chapel Hill, NC, USA; Nutrition Research Institute, University of North Carolina at Chapel Hill, Kannapolis, NC, USA; Department of Nutrition, Gillings School of Public Health, University of North Carolina at Chapel Hill, Chapel Hill, NC, USA; Lineberger Comprehensive Cancer Center, University of North Carolina at Chapel Hill, Chapel Hill, NC, USA

**Keywords:** metabolomics, vitamin D, gene–diet interaction, mouse, Collaborative Cross, lipid metabolism, amino acid metabolism, uremic toxemia

## Abstract

**Background:**

Liver metabolite concentrations have the potential to be key biomarkers of systemic metabolic dysfunction and overall health. However, for most conditions we do not know the extent to which genetic differences regulate susceptibility to metabolic responses. This limits our ability to detect and diagnose effects in heterogeneous populations.

**Objectives:**

Here, we investigated the extent to which naturally occurring genetic differences regulate maternal liver metabolic response to vitamin D deficiency (VDD), particularly during perinatal periods when such changes can adversely affect maternal and fetal health.

**Methods:**

We used a panel of 8 inbred Collaborative Cross (CC) mouse strains, each with a different genetic background (72 dams, 3–6/treatment group, per strain). We identified robust maternal liver metabolic responses to vitamin D depletion before and during gestation and lactation using a vitamin-D-deficient (VDD; 0 IU vitamin D_3_/kg) or -sufficient diet (1000 IU vitamin D_3_/kg). We then identified VDD-induced metabolite changes influenced by strain genetic background.

**Results:**

We detected a significant VDD effect by orthogonal partial least squares discriminant analysis (Q2 = 0.266, pQ2 = 0.002): primarily, altered concentrations of 78 metabolites involved in lipid, amino acid, and nucleotide metabolism (variable importance to projection score ≥1.5). Metabolites in unsaturated fatty acid and glycerophospholipid metabolism pathways were significantly enriched [False Discovery Rate (FDR) <0.05]. VDD also significantly altered concentrations of putative markers of uremic toxemia, acylglycerols, and dipeptides. The extent of the metabolic response to VDD was strongly dependent on genetic strain, ranging from robustly responsive to nonresponsive. Two strains (CC017/Unc and CC032/GeniUnc) were particularly sensitive to VDD; however, each strain altered different pathways.

**Conclusions:**

These novel findings demonstrate that maternal VDD induces different liver metabolic effects in different genetic backgrounds. Strains with differing susceptibility and metabolic response to VDD represent unique tools to identify causal susceptibility factors and further elucidate the role of VDD-induced metabolic changes in maternal and/or fetal health for ultimately translating findings to human populations.

## Introduction

Metabolomics provides a snapshot of metabolic processes at a particular time in a particular tissue of interest. Because the liver operates as one of the most important organs in nutrient and drug metabolism, the liver metabolome serves as an assessment of overall health. Using metabolomics, not only do we have the opportunity to identify biomarkers that may be indicative of health or disease status, but the high level of sensitivity provided by metabolomics studies allows a better understanding of the potential mechanisms of disease development (1). In fact, human studies have identified metabolic profiles that are proposed for use in diagnoses of diseases such as cardiovascular disease and diabetes (2–5).

Past research has demonstrated that genetic polymorphisms significantly contribute to the variance in divergent metabolic profiles in the human population (6–11). Genetic differences control protein expression, playing a direct role in metabolic enzyme concentrations/availability and downstream metabolite concentrations. Genetic profiles also serve as a major risk factor for the development of many health conditions. For example, in well-studied diseases such as breast cancer, specific genetic profiles are implicated in driving susceptibility to different forms of the disease in humans (12–14). Furthermore, specific metabolic phenotypes (metabotypes) related to disease progression have been defined (15). Collection and analysis of metabolomic data in genetically divergent populations with known genetic compositions such as in the study performed here allow the mechanistic link between the genetic component and the development of the potential disease to be further understood.

Environment, specifically diet, has a strong influence on the concentrations of circulating metabolites and metabolomic profiles (16). Nutrients directly entering the bloodstream from the gastrointestinal tract after a meal almost immediately affect serum metabolite concentrations. However, there is significant evidence that dietary patterns and long-term nutritional status can affect one's developed, consistent metabolic profile. For example, in rodent studies, high-fat diets have repeatedly been shown to alter fatty acid metabolites and β-oxidation pathways (17, 18). Human studies have also provided evidence that micronutrient intake affects metabolic profiles. Vitamin A, vitamin C, vitamin D, and vitamin E have been implicated in metabolic phenotypes ranging from enhanced oxidative stress to alterations in major macronutrient pathways (19–22). Although a growing number of studies are assessing the impact of diet on metabolomics, few studies have sufficiently addressed whether genetically divergent populations differ in terms of metabolomic responses. More importantly, the magnitude of genetically induced differences in the metabolome has rarely been assessed in diet studies, leaving it unclear whether genetics has a large or inconsequential impact on the final diet-induced metabolite concentrations and related health outcomes. Metabolomic studies from human and rodent research have evaluated metabolite concentrations across genetically divergent individuals and offer valuable insight on gene-by-environment interactions (23, 24).

This study focuses on the liver metabolomic impact of maternal vitamin D deficiency (VDD), a global health concern with some populations having VDD rates >80% (25). Maternal VDD has major implications for fetal and maternal health (26). It is well known that VDD impairs human fetal growth and skeletal and extraskeletal health (27–29). Epidemiological studies also implicate an emerging role for VDD in maternal health including increased risk of gestational diabetes mellitus and pre-eclampsia (30–34), but the molecular mechanisms remain unknown. Metabolomics presents an opportunity to elucidate molecular mechanisms of adverse maternal health outcomes of VDD and/or define new, previously undetermined, health consequences. For example, work suggests vitamin D status may also play a role in the metabolic regulation of pregnant women by modulating specific metabolites related to oxidative stress and inflammation (35). In addition, vitamin D supplementation in randomized clinical trials has been shown to beneficially alter metabolic biomarkers of obesity-related phenotypes (36). Overall, metabolomics research focusing on maternal vitamin D status, particularly during perinatal periods, is lacking. Providing a better understanding of maternal metabolic pathways that are altered by vitamin D status could provide important clues to the molecular mechanisms underlying maternal health, and potential biomarkers to assess efficacy of treatment.

The objective in this study was to identify robust metabolic signatures of VDD that are dependent on genetic background in order to *1*) identify maternal liver metabolites/metabolic pathways that are perturbed by VDD and could serve as candidate biomarkers of physiological changes; *2*) provide evidence for the first time that strain genetic background plays an important role in metabolic response to VDD; *3*) elucidate the extent to which strain genetic background influences metabolomic response to VDD; and *4*) identify strains that are particularly susceptible or resistant to major VDD-induced metabolic changes.

To determine the role of genetic background, we compared metabolic effects of chronic maternal VDD across 8 genetically divergent mouse strains of the Collaborative Cross (CC) (37). The CC is an inbred genetic reference population that was designed specifically to mimic the extent and distribution of genetic variation in human populations for the purpose of modeling how genetic differences influence molecular, physiological, and phenotypic differences for both normal and disease states (37). CC strains have a high number of naturally occurring (i.e., not artificially induced or engineered) DNA sequence differences across the genome, which represents >90% of the genetic variation among all inbred laboratory mouse strains when all lines are considered (37). However, CC strains are inbred and thus mice within a strain are highly genetically similar (up to 99% genetically identical; http://csbio.unc.edu/CCstatus). This allows for assessing biological replicates within the study by measuring effects on multiple mice from each strain. The combination of high genetic diversity between strains and genetic similarity within a strain is particularly useful for studying gene × environment interactions through repeated measures. The strain-specific VDD-altered metabolites and metabolic pathways identified here will help in future studies to more accurately assess the effects of VDD and identify susceptible populations.

## Methods

### Animals: housing, dietary treatment, and breeding scheme

Animal handling was performed in a humane and ethical manner in accordance with the Guide for the Care and Use of Laboratory Animals under the corresponding animal use protocol at the University of North Carolina (UNC) at Chapel Hill. CC inbred mouse strains CC001/Unc (CC001), CC011/Unc (CC011), CC051/TauUnc (CC051), CC041/TauUnc (CC041), CC004/TauUnc (CC004), CC017/Unc (CC017), CC032/GeniUnc (CC032), and CC042/GeniUnc (CC042) were obtained from the UNC Systems Genetics Core Facility (Chapel Hill, NC) (38). Vivarium temperature was maintained between 21 and 23°C with a 12-h light cycle. The light source (fluorescent bulbs) was not filtered, thus all animals were subjected to UV B exposure (wavelength: 280–315 nm) at a rate of 8.39E^−7^ W/cm^2^. Sterilized water and rodent unpurified diet were fed ad libitum.

**FIGURE 1:**
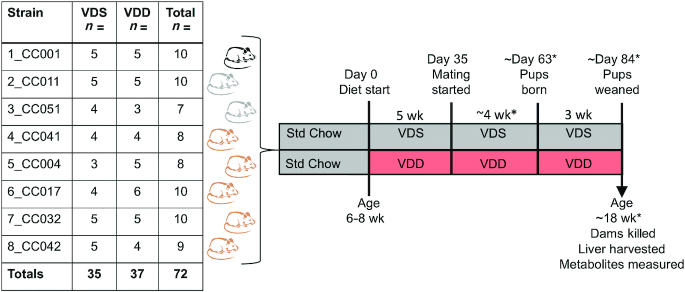
Treatment scheme and study design. Mice from 8 CC strains were treated for ∼12 wk (before, during, and after gestation) with either VDS diet (1000 vitamin D IU/kg) or VDD diet (0 IU vitamin D/kg). Before treatment, mice were on Std Chow: Teklad 8604 (2400 IU vitamin D/kg). Coat colors of CC strains are indicated by black (black), albino (gray), and agouti (brown). *n* = sample size. *Time/age varies based on time of conception. CC, Collaborative Cross; Std, standard; VDD, vitamin D deficient; VDS, vitamin D sufficient.

As **Figure 1** shows, we assessed the impact of chronic VDD during pregnancy and lactation on postweaning maternal vitamin D status across an experimental population of 8 CC inbred mouse strains. Virgin females 6–8 wk old were treated with AIN-93G purified rodent diet with normal calcium and phosphorus concentrations that was either vitamin D sufficient [VDS; DYET#110700 containing 1000 IU vitamin D_3_/kg, Dyets Inc. (39)] or vitamin D deficient [VDD; DYET#119266, identical to #110700 (39) except containing vitamin-free casein and vitamin mix #3199255 to achieve 0 IU vitamin D_3_/kg, Dyets Inc.]. Assuming mean dietary intake of 3–5 g feed/d, VDS mice consumed ∼3–5 IU vitamin D/d, which meets the recommended daily intake for laboratory mice (40). VDD mice received no vitamin D throughout the treatment period. AIN-93G provides 14%, 32%, and 54% calories from fat, protein, and carbohydrate, respectively. Dams remained on the diet for ∼12 wk including 5 wk before mating, ≥1 wk during mating, 3 wk during gestation, and 3 wk during lactation. Dams that did not get pregnant or did not maintain pups to weaning were removed from the study. To minimize pup lethality due to cannibalism or neglect, litter size was only recorded at weaning. Metabolomics of pups and sires were not assessed in this study. All dams in this study were killed while still on experimental diets (VDS and VDD) at pup weaning at ∼18 wk on average. All mice were killed by carbon dioxide exposure in accordance with current recommendations by the 2020 American Veterinary Medical Association guidelines (www.avma.org).

### Quantification of plasma 25-hydroxyvitamin D

Whole blood was collected by cardiac puncture immediately after killing the animals. Plasma was prepared from heparin-treated blood and snap-frozen in liquid nitrogen. 25-Hydroxyvitamin D [25(OH)D] concentrations were measured in the UNC Nutrition Obesity Research Center Metabolic Molecular Phenotyping Core using ELISA (Eagle Biosciences).

### Liver metabolite measurements

At weaning, dams were killed and their livers flash frozen, pulverized, divided into aliquots, and sent for metabolomics analyses (Metabolon, Inc.). Sample preparation was conducted as previously described (41). Untargeted metabolic profiling of maternal whole liver samples was determined using reverse phase ultra-performance liquid chromatography-tandem mass spectrometry (RP/UPLC-MS/MS) or hydrophilic interaction liquid chromatography ultra-performance liquid chromatography-tandem mass spectrometry (HILIC/UPLC-MS/MS). The following 4 platforms were used to capture metabolites with a wide range of chemical properties in the metabolome: *1*) RP/UPLC-MS/MS with acidic positive ion mode electrospray ionization (ESI) for hydrophilic compounds, *2*) RP/UPLC-MS/MS with acidic positive ion mode ESI for hydrophobic compounds, *3*) RP/UPLC-MS/MS with basic negative ion mode ESI, and *4*) HILIC/UPLC-MS/MS with negative ion mode ESI. Metabolites were identified by comparison to a library of entries generated by Metabolon containing the *m/z*, retention time/index, and chromatographic data (including tandem MS spectral data). Metabolite names ending in an asterisks (*) indicate compounds that have not been officially confirmed based on a standard. Quality control of metabolomics data to account for instrument and process variability was conducted as previously described and met the acceptance criteria of Metabolon (42).

### Metabolomics data preprocessing

To account for interday differences in instrument tuning, raw data were normalized by shifting the median of the data for each day to 1 and correcting each compound proportionately. Data for each compound were then rescaled to set the median to 1. This maintains variation between samples in the data while placing compounds with widely different numerical ranges on the same scale to make them directly comparable and of equal weighting. This prevents compounds with a large numerical range from masking compounds with smaller ranges that may be indicative of class differences and therefore significant to the multivariate analysis model. Missing values for samples falling below the limit of detection were imputed with the minimum observed value for a metabolite.

### Multiclass orthogonal partial least squares discriminant analysis to identify metabolites altered by strain and diet

#### Strain effects

Orthogonal partial least squares discriminant analysis (OPLS-DA) for assessing strain effects on metabolite concentrations was performed in SIMCA 14.1 software (Umetrics). The variable importance to projection (VIP) score for each metabolite was calculated to quantitatively represent metabolite feature importance in the fitted model. The VIP score algorithm is copyright protected by SIMCA (43).

#### Diet effects

Ordinary least squares linear regressions for each metabolite (metabolite ∼ diet + strain) were fit using the lm() function in R (44) with both predictors being categorical variables. The metabolite concentrations were then corrected for strain effects by subtracting the regression coefficients for strain from the corresponding response variable, except for the regression baseline strain CC011 for which the response variable remained the same with correction. OPLS-DA was carried out on the strain-corrected metabolite data using the R package “ropls (ver 1.16.0).” Statistical significance of diet separation was assessed through comparing model metrics (*R*^2^ Y and *Q*^2^) with those generated from 1000 random permutations to generate the pQ2 value. Root mean squares of the orthogonal and predictive VIP scores were calculated to represent the importance of the metabolites in the model.

### MetaboAnalyst pathway analyses

Metabolite Set Enrichment Analyses (MSEA) were performed on all metabolites with a VIP ≥1.5 that matched the database using the “Pathway-associated metabolite sets (SMPDB)” database in the MetaboAnalyst software (https://www.metaboanalyst.ca). Pathway analysis was performed using the “Mus musculus (KEGG)—Previous” database in the MetaboAnalyst software. Metabolon-derived names were converted to names recognized by MetaboAnalyst (**Supplemental Tables 1**A, **2**A, **3**A, **4**A, **5**A). Lipid metabolite names were poorly recognized and thus these analyses are likely an underrepresentation of the effects on lipid metabolism (see Discussion). Supplemental Tables 1B, 2B, 3B, 4B, and 5B present pathway results.

### Other statistical analyses

Statistical analyses were performed in JMP Pro software version 12.2.0 (SAS Institute). Where applicable, normality of data distribution was confirmed by the Shapiro–Wilk goodness-of-fit test and variance tested by Bartlett's test. Data that met assumptions of normality were analyzed by parametric tests (*t* test, ANOVA, linear regression), whereas data that did not meet assumptions of normality were analyzed by nonparametric tests (Wilcoxon/Kruskal–Wallis test) as indicated in each figure legend. Data with normal distributions but unequal variance were analyzed by Welch's *t* test/ANOVA as indicated. Where applicable, the Tukey–Kramer honestly significant difference (HSD) post hoc test was used to determine which strains in the group were significantly different from the rest. For all comparisons, *P* values < 0.05 were considered statistically significant. Error bars represent standard error of the mean (SEM) for all bar graphs. Summary values shown in text represent the mean ± SEM.

## Results

### VDD reduced plasma 25(OH)D concentrations across all strains

**FIGURE 2:**
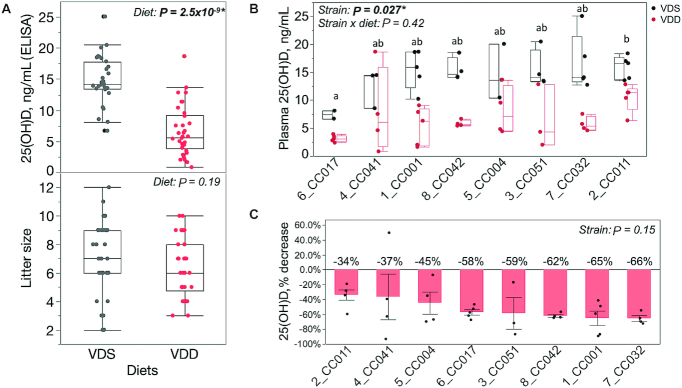
Strain-specific maternal plasma vitamin D status with and without dietary vitamin D depletion. Maternal plasma 25(OH)D concentrations measured by ELISA. (A) Box and whiskers plot of maternal 25(OH)D (top panel) and litter sizes (bottom panel) for each diet (all strains combined). Each dot represents a single female. Main effect of diet *P* value shown as determined by Wilcoxon test [25(OH)D] or 2-sided *t* test (litter size). (B) Box and whiskers plot of maternal 25(OH)D concentrations separated by strain. Samples sizes are as follows for VDS and VDD samples for each strain in order from left to right: *n* = 2,5 (CC017); 3,4 (CC041); 5,5 (CC001); 4,4 (CC042); 3,4 (CC004); 4,3 (CC051); 5,4 (CC032); 5,5 (CC011). *x*-Axis denotes strain name. Main effect of strain and strain × diet *P* value shown as determined by linear regression (*y* = strain + diet + strain × diet). Letters denote strains that differ significantly (a ≠ b = ab) as determined by Tukey's honestly significant difference (HSD) post hoc test. (C) Percentage reduction in maternal plasma 25(OH)D concentration caused by VDD [(VDD^Sample^ − VDS^Mean^)/VDS^Mean^], separated by strain. Main effect of strain *P* value shown as determined by Welch's ANOVA. Mean 25(OH)D percentage reduction values for each strain listed above each bar. Asterisks (*) and bold font indicate statistically significant *P* values. CC, Collaborative Cross; VDD, vitamin D deficient; VDS, vitamin D sufficient; 25(OH)D, 25-hydroxyvitamin D.

We assessed the impact of chronic VDD during pregnancy and lactation on postweaning maternal vitamin D status across an experimental population of 8 CC inbred mouse strains (Figure 1). To confirm that dietary vitamin D depletion for ∼12 wk decreases circulating vitamin D metabolite status in this experimental population, total plasma 25(OH)D concentrations for dams treated with VDS diet were compared with those for dams treated with VDD diet. As expected, VDD dams had significantly lower 25(OH)D concentrations than VDS dams (∼56% lower on average) (**Figure 2**A). Despite the reduction in dietary vitamin D availability during pregnancy there was no significant difference in litter size between diet groups (Figure 2A). After adjustment for diet, there remained a significant strain effect on plasma 25(OH)D concentrations for all samples combined (Figure 2B). Strain CC017 exhibited the lowest mean 25(OH)D concentrations for both diet groups (VDS: 7.4 ± 1.01; VDD: 3.2 ± 0.60 ng/mL), which were significantly lower than the 25(OH)D concentrations for CC011 (VDS: 15.9 ± 2.1 ng/mL; VDD: 10.5 ± 2.5 ng/mL) (Figure 2B). The extent of 25(OH)D depletion caused by VDD (percentage decrease) did not differ significantly among the strains although it varied from −34% (CC011) to −66% (CC032) (Figure 2C).

### CC maternal liver metabolomic profiles were determined by strain

**FIGURE 3:**
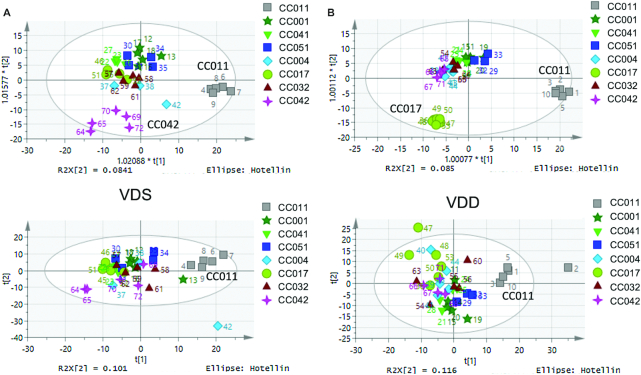
Strain effects on dam postweaning liver metabolomic profiles. Orthogonal partial least squares discriminant analysis (top row) and Principal Components Analysis (PCA) (bottom row) plots for strain stratified by diet: (A) VDS, (B) VDD. Each marker color and shape denotes a different strain. CC, Collaborative Cross; VDD, vitamin D deficient; VDS, vitamin D sufficient.

To assess effects of VDD on maternal liver metabolomics profiles, we performed untargeted global metabolic profiling via MS through Metabolon, Inc. on dam livers from the VDS and VDD treatment groups (Figure 1 lists the sample sizes). We first examined whether there was a significant strain effect on liver concentrations of the 654 metabolites detected using OPLS-DA applied to samples stratified by diet. Most VDS-treated strains had similar liver metabolomics profiles (based on clustering), but strains CC011 and CC042 clustered separately from others (**Figure 3**A). VDD-treated samples exhibited similar strain clustering results including the distinct profile of CC011. However, the distinctive metabolic signature of CC042 was no longer present and a new signature was detected for CC017 (Figure 3B). To identify metabolites driving these strain-specific metabolic signatures, we performed OPLS-DA analyses supervising each model with the following variables: for VDS-treated samples, CC011 compared with other strains (VDS-CC011 signature) and CC042 compared with other strains (VDS-CC042 signature); and for VDD-treated samples, CC011 compared with other strains (VDD-CC011 signature) and CC017 compared with other strains (VDD-CC017 signature).

**FIGURE 4:**
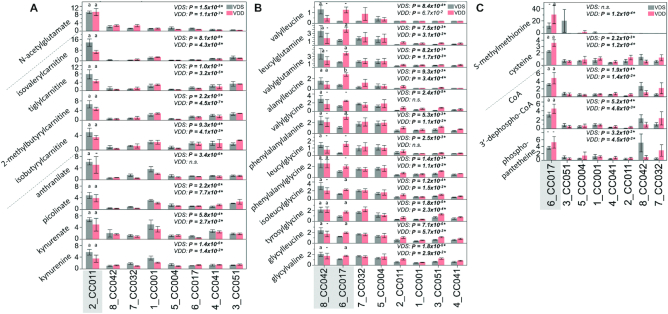
Metabolite markers of strain-specific metabolomic profiles. Bar graphs with SEMs of metabolite concentrations (*y*-axis) that make up strain (*x*-axis) metabolomic signatures. (A) VDS-CC011 and VDD-CC011 signature metabolites; (B) VDS-CC042 signature metabolites; (C) VDD-CC017 signature metabolites. Asterisks (*) and bold font indicate statistically significant *P* values. n.s., not statistically significant (*P* > 0.05). Dashed line separates metabolites in different metabolic pathways. The letter above a bar indicates whether the strain of interest (shaded in gray) is significantly different (a/b) or not significantly different (-) from ≥1 strain in the group as determined by Tukey's honestly significant difference (HSD) post hoc test. CC, Collaborative Cross; VDD, vitamin D deficient; VDS, vitamin D sufficient.

The VDS-CC011 signature was primarily defined by 80 metabolites selected by VIP ≥1.5 (Supplemental Table 1A). Using MSEA in the MetaboAnalyst (45) software, we determined potential enrichment for metabolites in the glucose–alanine cycle [*P* = 0.009, False Discovery Rate (FDR) >0.5], α-linolenic acid and linoleic acid metabolism (*P* = 0.037, FDR >0.5), and the urea cycle (*P* = 0.045, FDR >0.5) (Supplemental Table 1B). Because the FDR was >0.5 for these pathways we did not consider these results statistically significant. Among the top 20 metabolites with the greatest fold difference between VDS-CC011 and other VDS-treated strains were 4 acylcarnitines, 4 metabolites involved in tryptophan metabolism, and N-acetylglutamate. All were substantially elevated in VDS-CC011 samples (**Figure 4**A).

For the VDD-CC011 signature, we identified 70 metabolites with VIP ≥1.5 (Supplemental Table 2A). Not surprisingly, the VDD-CC011 signature included 41 metabolites also found in the VDS-CC011 signature. Similarly, this included potential enrichment for metabolites involved in α-linolenic acid and linoleic acid metabolism (*P* = 0.023, FDR >0.5), the urea cycle (*P* = 0.025, FDR >0.5), and ammonia recycling (*P* = 0.037, FDR >0.5) (Supplemental Table 2B). Again, because the FDR was >0.5 for these pathways we did not consider these results statistically significant. Acylcarnitines, tryptophan metabolites, and N-acetylglutamate were again among the top 20 metabolites when ranked by fold change. These were significantly elevated in VDD-CC011 samples compared with other strains (Figure 4A).

For the VDS-CC042 signature, we identified 63 metabolites with VIP ≥1.5 (Supplemental Table 3A). Although no metabolic pathways showed potential enrichment (*P *< 0.05) (Supplemental Table 3B), we observed a high number (12) of upregulated dipeptides (Figure 4B). Further assessment showed these dipeptides were seemingly also elevated in VDD-CC042 samples but partially masked from being a unique signature of VDD-CC042 owing to upregulation of several other VDD-treated strains, most notably CC017 and CC051 (Figure 4B).

The presence of a VDD-CC017 signature represents VDD-induced metabolite concentrations unique to the CC017 genetic background, suggesting that this strain may be particularly metabolically responsive to VDD compared with other strains. We identified 66 metabolites for the VDD-CC017 signature with VIP ≥1.5 (Supplemental Table 4A), including potential enrichment for metabolites involved in pantothenate (vitamin B-5) and CoA biosynthesis (*P* = 0.005, FDR >0.5) and glutathione metabolism (*P* = 0.027, FDR >0.5) (Supplemental Table 4B). Because the FDR was >0.5 for these pathways we did not consider these results statistically significant. Among the top 20 metabolites with the greatest fold difference between VDD-CC017 and other VDD-treated strains were metabolites derived from dipeptides (Figure 4B), one-carbon metabolism, and vitamin B-5 and CoA metabolism (Figure 4C). Concentrations of these metabolites were slightly higher in VDD-treated CC017 mice than in VDS-treated mice but not statistically significant (Figure 4C). Therefore, these metabolite changes were strain specific but likely independent of diet. Similarly, 2 metabolites derived from one-carbon metabolism and 3 metabolites involved in vitamin B-5 and CoA metabolism were higher in both VDS- and VDD-treated CC017 samples (Figure 4C). On the other hand, dipeptide concentrations were higher in VDD-treated CC017 samples than in other strains and VDS-treated CC017 samples, representing a strain-specific diet effect (Figure 4B).

### VDD-induced CC maternal liver metabolomic profiles were masked by strain effects

**FIGURE 5:**
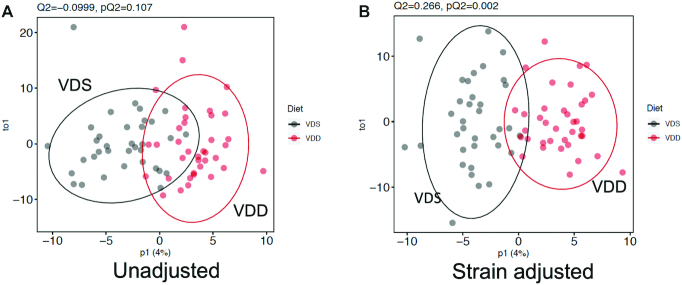
Diet effects on dam postweaning liver metabolomic profiles. Orthogonal partial least squares discriminant analysis plots for strain stratified by diet: (A) metabolite concentrations run without strain adjustment; (B) residuals of metabolites run after strain adjustment (see Methods). Marker color denotes diet. Ellipses denote 95% CIs. VDD, vitamin D deficient; VDS, vitamin D sufficient.

**FIGURE 6:**
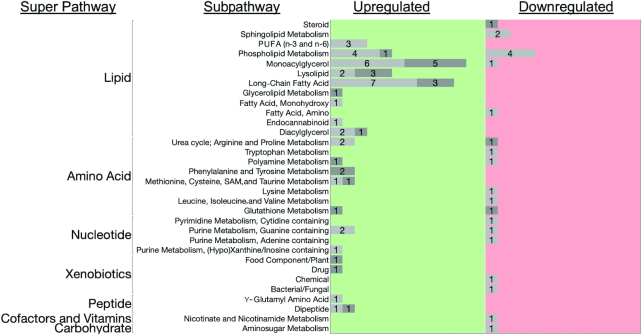
Liver metabolic pathways altered by chronic VDD. Bar graph of the number of liver metabolites altered by VDD for each super- and subpathway. For each metabolic subpathway, the number of affected metabolites are listed in the corresponding bar. Metabolites with <1.5-fold change in concentrations are shown as light gray bars and those with ≥1.5-fold change in concentrations are shown as dark gray bars. Left panel (green) shows number of metabolites that increased with VDD. Right panel (red) shows metabolites that decreased with VDD. VDD, vitamin D deficiency.

To determine the extent to which strain effects on metabolite concentrations might mask the effects of diet in this genetically divergent population (as they might confound a standard metabolomic analysis that focused on diet), we assessed the effect of diet with and without adjustment for strain. As expected, OPLS-DA analyses of VDD effects before adjustment for strain did not result in significant separation of sample metabolic profiles by diet (pQ2 = 0.11) (**Figure 5**A). However, after adjustment for strain (see Methods), we detected a significant diet effect on metabolite profiles (pQ2 = 2.2 × 10^−3^) (Figure 5B). Owing to this confounding by strain of diet effects in this population of mice, we assessed diet effects by OPLS-DA only after adjustment for strain effects. Using a VIP threshold of ≥1.5, we detected 78 VDD-altered liver metabolites (Supplemental Table 5A) primarily upregulated and involved in lipid, amino acid, and nucleotide metabolism (**Figure 6**).

#### VDD disrupted maternal liver metabolism of fatty acids and glycerophospholipids in a strain-dependent manner

**FIGURE 7:**
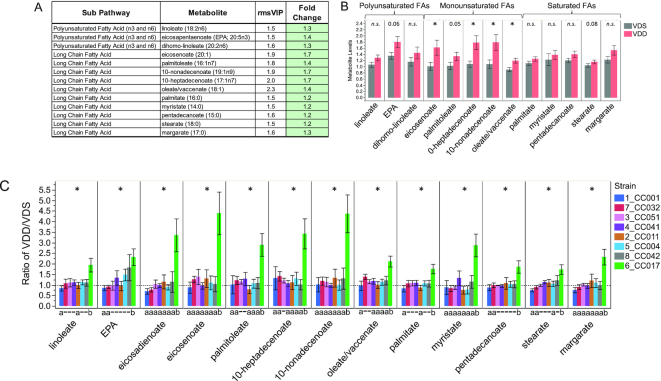
Strain-specific effect of VDD on liver concentrations of fatty acids. (A) Metabolite names, metabolic subpathways, fold changes, and VIP scores. Green fold change cell indicates increased concentrations in VDD samples compared with VDS. (B) Bar graphs and SEMs of liver concentrations of metabolites by diet. *P < 0.05; *P *< 0.1 shown above bar, determined by Wilcoxon test. n.s., not statistically significant (*P*  > 0.05). (C) Bar graph of mean change in metabolite concentrations calculated as ratio of VDD/VDS. Dotted line indicates mean metabolite concentrations of VDS samples used as relative control values for calculating VDD response. Mean values above dotted line indicate increased metabolite concentrations in VDD samples compared with VDS; Mean values below dotted line indicate decreased metabolite concentrations in VDD compared with VDS. *Strain effect *P *< 0.05 as determined by ANOVA. Letters (a and b) below graphs indicate strains with significantly different VDD-induced fold change in metabolite concentrations compared with other strains in the group as determined by Tukey's honestly significant difference (HSD) post hoc test. CC, Collaborative Cross; EPA, eicosapentaenoate; rmsVIP, root measure of the orthogonal and predictive variable importance to projection; VDD, vitamin D deficient; VDS, vitamin D sufficient.

Using Metaboanalyst (45), we determined that for the 78 metabolites altered by VDD (VIP ≥1.5) there was significant enrichment for metabolites in pathways for biosynthesis of unsaturated fatty acids (*P* = 6.4 × 10^−5^, FDR = 5.2 × 10^−3^) and glycerophospholipid metabolism (*P* = 8.2 × 10^−4^, FDR = 3.3 × 10^−2^) and potential enrichment for linoleic acid metabolism (*P* = 9.3 × 10^−3^, FDR = 0.25), fatty acid biosynthesis (*P* = 2.3 × 10^−2^, FDR = 0.42), and arginine and proline metabolism (*P* = 2.5 × 10^−2^, FDR = 0.42) (Supplemental Table 5B). Of the 13 metabolites involved in biosynthesis of unsaturated fatty acids (**Figure 7**A), after adjustment for strain, only eicosenoate (20:1), 10-heptadecenoate (17:1n–7), 10-nonadecenoate (19:1n–9), and oleate/vaccinate (18:1) were determined to have significantly higher concentrations for VDD dams (Figure 7B). Stratification by strain revealed the population-level effect for this pathway was primarily driven by the response of a single strain (CC017), with small effect size (if any) contributions from other strains. There was a significant strain effect on the fold change of all 13 fatty acid metabolite concentrations in response to VDD treatment, with CC017 mice seemingly the most responsive to VDD-induced changes in fatty acid metabolism, exhibiting significantly and substantially (≤4.5-fold) higher concentrations for all 13 metabolites (Figure 7C). Other strains were less responsive (<2-fold) (Figure 7C).

**FIGURE 8:**
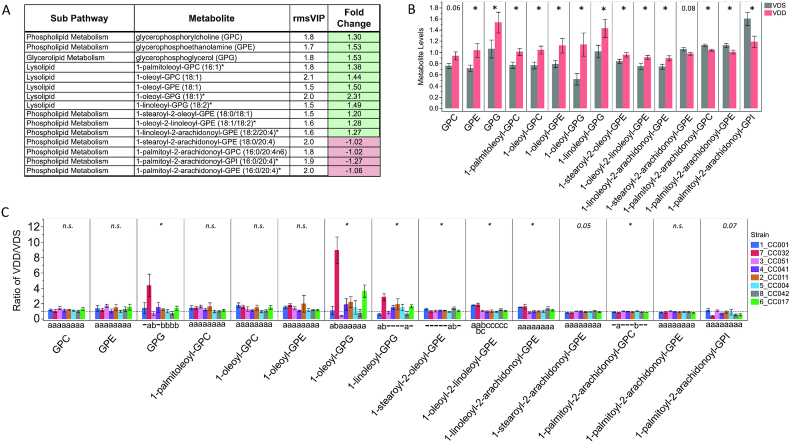
Strain-specific effect of VDD on liver concentrations of glycerophospholipid metabolites. (A) Metabolite names, metabolic subpathways, fold changes, and VIP scores. Green fold change cell indicates increased concentrations in VDD samples compared with VDS. (B) Bar graphs and SEMs of liver concentrations of metabolites by diet. *P < 0.05; *P *< 0.1 shown above bar, determined by Wilcoxon test. (C) Bar graph of mean change in metabolite concentrations calculated as ratio of VDD/VDS. Dotted line indicates mean metabolite concentrations of VDS samples used as relative control values for calculating VDD response. Mean values above dotted line indicate increased metabolite concentrations in VDD samples compared with VDS; Mean values below dotted line indicate decreased metabolite concentrations in VDD compared with VDS. *Strain effect *P *< 0.05 as determined by ANOVA. n.s., not statistically significant (*P*  > 0.05). Letters (a and b) below graphs indicate strains with significantly different VDD-induced fold change in metabolite concentrations compared with other strains in the group as determined by Tukey's honestly significant difference (HSD) post hoc test. CC, Collaborative Cross; GPC, glycerophosphorylcholine; GPE, glycerophosphoethanolamine; GPG, glycerophosphoglycerol; GPI, glycerophosphoinositol; rmsVIP, root measure of the orthogonal and predictive variable importance to projection; VDD, vitamin D deficient; VDS, vitamin D sufficient.

For metabolites involved in glycerophospholipid metabolism with VIP ≥1.5, VDD-treated dams exhibited increased concentrations of 11 metabolites and decreased concentrations of 4 metabolites (**Figure 8**A). Concentrations of 13 of the 15 metabolites were determined to be significantly altered by VDD (Figure 8B). After stratification by strain, a similar directional trend of response to VDD was detected for most strains (Figure 8C). We determined a significant difference among strains in the fold change of most of the glycerophospholipid metabolites, in this case driven mostly by changes in strain CC032 (Figure 8C). Overall, CC032 mice were seemingly the most responsive to VDD-induced glycerophospholipid metabolite changes, with the greatest number of metabolites with significant changes, including an ∼9-fold increase in 1-oleoyl-glycerophosphoglycerol concentrations (Figure 8C).

#### VDD had a robust impact on maternal liver metabolites that are putative biomarkers of uremic toxemia, lipid metabolites, and dipeptide metabolites in a strain-dependent manner

**FIGURE 9:**
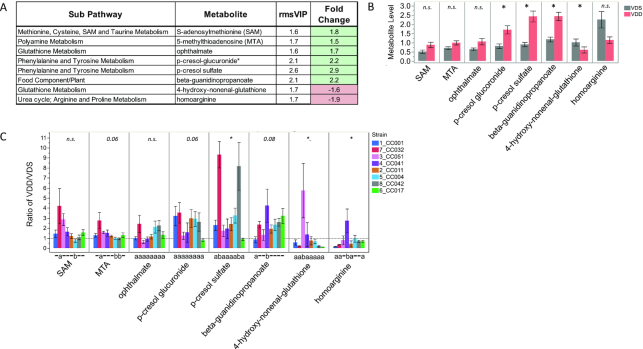
Strain-specific effect of VDD on liver concentrations of putative biomarkers of uremic toxemia. (A) Metabolite names, metabolic subpathways, fold changes, and VIP scores. Green fold change cell indicates increased concentrations in VDD samples compared with VDS. (B) Bar graphs and SEMs of liver concentrations of metabolites by diet. *P < 0.05, determined by Median test. (C) Bar graph of mean change in metabolite concentrations calculated as ratio of VDD/VDS. Dotted line indicates mean metabolite concentrations of VDS samples used as relative control values for calculating VDD response. Mean values above dotted line indicate increased metabolite concentrations in VDD samples compared with VDS; Mean values below dotted line indicate decreased metabolite concentrations in VDD compared with VDS. *Strain effect *P *< 0.05 as determined by Welch's ANOVA. Letters (a and b) below graphs indicate strains with significantly different VDD-induced fold change in metabolite concentrations compared with other strains in the group as determined by Tukey's honestly significant difference (HSD) post hoc test. CC, Collaborative Cross; MTA, S-methylthioadenosine; n.s., not statistically significant (*P* > 0.05); rmsVIP, root measure of the orthogonal and predictive variable importance to projection; SAM, S-adenosylmethionine; VDD, vitamin D deficient; VDS, vitamin D sufficient.

**FIGURE 10:**
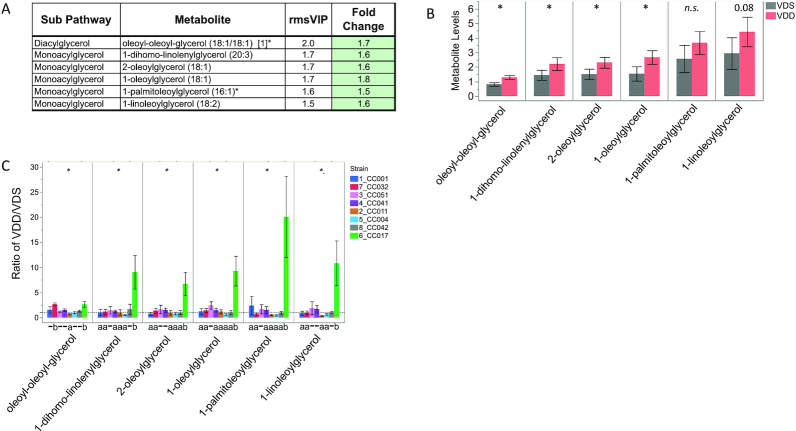
Strain-specific effect of VDD on liver concentrations of acylglycerols. (A) Metabolite names, metabolic subpathways, fold changes, and VIP scores. Green fold change cell indicates increased concentrations in VDD samples compared with VDS. (B) Bar graphs and SEMs of liver concentrations of metabolites by diet. *P < 0.05; *P *< 0.1 shown above bar, determined by Wilcoxon test. n.s., not statistically significant (*P*  > 0.05). (C) Bar graph of mean change in metabolite concentrations calculated as ratio of VDD/VDS. Dotted line indicates mean metabolite concentrations of VDS samples used as relative control values for calculating VDD response. Mean values above dotted line indicate increased metabolite concentrations in VDD samples compared with VDS; Mean values below dotted line indicate decreased metabolite concentrations in VDD compared with VDS. *Strain effect *P *< 0.05 as determined by ANOVA. Letters (a and b) below graphs indicate strains with significantly different VDD-induced fold change in metabolite concentrations compared with other strains in the group as determined by Tukey's honestly significant difference (HSD) post hoc test. CC, Collaborative Cross; rmsVIP, root measure of the orthogonal and predictive variable importance to projection; VDD, vitamin D deficient; VDS, vitamin D sufficient.

Twenty-five of the 78 VDD-altered metabolites identified by OPLS-DA exhibited a robust change (≥1.5-fold) (Supplemental Table 5A). These included 8 uremic solutes [implicated as biomarkers of uremic toxemia (38)] (**Figure 9**A), 6 acylglycerols (**Figure 10**A), and 8 glycerophospholipids (Figure 8A) (as aforementioned).

Concentrations of 3 uremic solutes were determined to be significantly increased by VDD and 1 significantly decreased (Figure 9B). After stratification by strain, a similar directional trend of uremic solute response to VDD was detected for most strains; however, there was a significant difference among the strains in the fold change in concentrations for several metabolites (Figure 9C). Strain CC032 seemingly was the main driver of VDD-increased uremic solute metabolite concentrations including an ∼9-fold increase in p-cresol sulfate (Figure 9C). Interestingly, the 2 VDD-decreased uremic solutes exhibited strain differences driven by increased concentrations in CC051 (4-hydroxy-nonenal-glutathione) and CC041 (homoarginine), demonstrating that strains also have an inverse response to VDD for some metabolites (Figure 9C).

Four acylglycerol metabolites were determined to be significantly increased by VDD, although all 6 followed a similar trend of increased concentrations (Figure 10B). Similarly to the strain-specific VDD-induced changes in fatty acid metabolism (Figure 7C), stratification by strain revealed that the population-level effect for acylglycerols was also primarily driven by the response of CC017, with only small effect size contributions from other strains (Figure 10C). CC017 mice were the most responsive to VDD for this pathway, with fold changes significantly different from other strains, including ∼10-fold increase in 1-linoleoylglycerol (Figure 10C).

**FIGURE 11:**
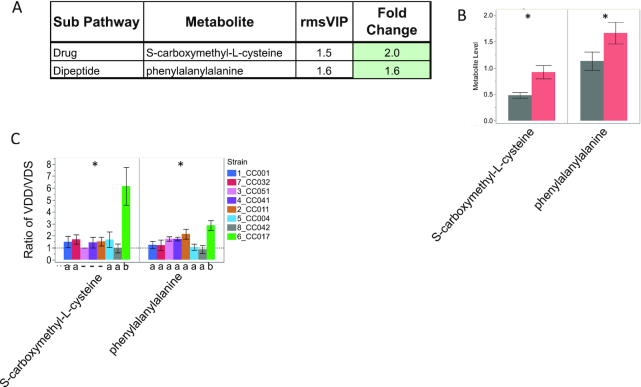
Strain-specific effect of VDD on liver concentrations of phenylalanylalanine and S-carboxymethyl-l-cysteine. (A) Metabolite names, metabolic subpathways, fold changes, and VIP scores. Green fold change cell indicates increased concentrations in VDD samples compared with VDS. (B) Bar graphs and SEMs of liver concentrations of metabolites by diet. *P < 0.05, determined by Wilcoxon test. (C) Bar graph of mean change in metabolite concentrations calculated as ratio of VDD/VDS. Dotted line indicates mean metabolite concentrations of VDS samples used as relative control values for calculating VDD response. Mean values above dotted line indicate increased metabolite concentrations in VDD samples compared with VDS; Mean values below dotted line indicate decreased metabolite concentrations in VDD compared with VDS. *Strain effect *P *< 0.05 as determined by ANOVA. Letters (a and b) below graphs indicate strains with significantly different VDD-induced fold change in metabolite concentrations compared with other strains in the group as determined by Tukey's honestly significant difference (HSD) post hoc test. CC, Collaborative Cross; rmsVIP, root measure of the orthogonal and predictive variable importance to projection; VDD, vitamin D deficient; VDS, vitamin D sufficient.

We also detected a ≥1.5-fold change in 1 dipeptide (phenylalanylalanine) and 1 cysteine derivative (S-carboxymethyl-l-cysteine) (**Figure 11**A), both of which were significantly increased in VDD-treated samples (Figure 11B). For both metabolites, CC017 was the most responsive strain with fold changes significantly higher than for other strains, including an ∼6-fold change in S-carboxymethyl-l-cysteine (Figure 11C). This CC017-specific response to VDD is in addition to the VDD-induced increase in dipeptides shown in Figure 4B.

## Discussion

This study used a combination of comparative approaches to demonstrate that liver metabolic response to VDD is strongly determined by genetic background. In this genetically divergent population of CC strains, VDD induced significant changes in maternal liver metabolism of fatty acids, glycerophospholipids, putative biomarkers of uremic toxemia, acylglycerols, and dipeptides. Interestingly, all of these effects were highly strain dependent, with 1 or 2 strains at most robustly affected whereas other strains showed mild or no effects. In addition to strain differences in extent of susceptibility to VDD-induced metabolic changes, different strains also exhibited dysregulation of different metabolic pathways in response to VDD. It remains to be determined whether these VDD-induced metabolite changes are detrimental or protective responses.

Although all strains tested exhibited similar (not significantly different) depletion of 25(OH)D when placed on VDD diet, there was a significant strain effect on overall 25(OH)D concentrations. We identified several strains that were particularly susceptible to VDD-induced changes in specific metabolic pathways. CC017 exhibited a strain-specific metabolomic signature when exposed to VDD that included upregulation of metabolites involved in dipeptides, one-carbon metabolism, vitamin B-5 and CoA metabolism, fatty acid synthesis, acylglycerol, and cysteine metabolism. These could be indicators of severely impaired fatty acid metabolism, which has been previously demonstrated for VDD rodent studies (46).

Compared with other strains, CC017 had the lowest mean concentrations of basal (VDS) circulating 25(OH)D and exhibited on average ∼58% depletion in 25(OH)D when placed on VDD diet. In contrast, CC032, the strain with the highest mean concentrations of circulating 25(OH)D for VDS samples and on average ∼66% depletion of 25(OH)D on VDD diet, also exhibited a strain-specific VDD signature. However, CC032 was instead more susceptible to VDD-altered metabolism of glycerophospholipids and uremic solutes implicated in uremic toxemia. Increased concentrations of uremic solutes in the blood implicate impaired kidney function and failure to excrete potentially toxic metabolites that could build up and go back into the circulation and cause illness (47). The consequences of increases of these metabolites in the liver are unclear because they have primarily been measured in blood and urine.

We have also identified several new strain-specific liver metabolomic profiles that were independent of diet and can be used to define the basal metabolic status of these CC strains. Compared with other strains, VDS- and VDD-treated CC011 mice exhibited higher concentrations of acylcarnitines, tryptophan metabolites, and N-acetylglutamate. Acylcarnitines help transport fatty acids into the mitochondria for β-oxidation and upregulation could indicate increased β-oxidation for energy production (48). Increased acylcarnitines are biomarkers of metabolic disease such as obesity and diabetes (49). Tryptophan is a key upstream precursor of several important neurotransmitters, including serotonin. However, the tryptophan metabolites detected in this study are generated in an alternate pathway and have anti-inflammatory properties. Upregulation of anti-inflammatory tryptophan metabolites has been proposed to divert tryptophan from neurotransmitter-generating pathways, an outcome associated with intestinal inflammation (50). CC011 mice exhibit spontaneous colitis (51). Although the mice assessed here are younger than the previously shown age of onset, these could represent early markers of the disease. N-acetylglutamate is synthesized in the mitochondria and is a key metabolite in the liver urea cycle responsible for activating carbamoyl phosphate synthetase 1 in mitochondria (51, 52). High N-acetylglutamate could indicate increased amino acid catabolism. Another strain effect independent of diet was found for VDS-treated CC042 mice which had higher concentrations of dipeptides than other strains. The cause is unclear but it could be indicative of incomplete protein catabolism.

This study has provided novel and highly valuable information to our understanding of the role of gene × diet interactive effects on liver metabolic processes. However, the relatively small sample size limits power to detect metabolite changes and thus what is presented here is an underrepresentation of the true magnitude of the effect of VDD on the metabolome. We also focused primarily on robust differences in metabolite concentrations (VIP ≥1.5 and fold change ≥1.5), which means many more subtle changes were not explored. This was done for the purpose of limiting false-positive outcomes due to our limited sampling of the populations. As noted in the Methods, lipid metabolite names are particularly challenging to map to currently available databases owing to the wide variety in nomenclature. Many of our Metabolon-designated lipid metabolites were not found in the MetaboAnalyst databases used and therefore we were likely unable to completely define the true impact of VDD on lipid metabolism in the liver. As database matching improves, this limitation will become less of an issue.

It is important to point out that many of these VDD-induced metabolic changes would likely not have been possible to detect in this genetically divergent population without the ability to adjust for strain effects. This brings to question whether many metabolomic effects are confounded for genetic background and either go undetected in populations where differences are unidentified (false negatives) or are false positives for a treatment effect when they are actually caused by genetic differences. This study provides a premise for further investigating this potential issue and highlights the need for studies that integrate the impacts of genetic and environmental differences on the metabolome.

Taken together, these data show that VDD can have substantial effects on maternal liver metabolism, particularly disrupting lipid and amino acid metabolism. Furthermore, these responses to VDD are strain specific, possibly due to genetic differences in enzymes responsible for the metabolic pathways affected. Our finding of VDD-induced upregulation of several uremic solutes that are putative biomarkers of uremic toxemia demonstrates the potential for VDD to affect liver and/or kidney function in a manner that could adversely affect maternal and fetal health. Further studies are necessary to identify the genes and genetic variants responsible for the strain differences and to determine the consequences of upregulation of these potentially toxic metabolites.

## Supplementary Material

nzaa106_Supplemental_File

## Notes

Supported by National Institute of Environmental Health Sciences Transition to Independent Environmental Health Research Career Development Award KES023849A (to FYI), pilot funding from the University of North Carolina at Chapel Hill Nutrition Research Institute (to FYI), and National Institute of General Medical Sciences grant R35GM127000 (to WV).

Author disclosures: the authors report no conflicts of interest.

Supplemental Tables 1–5 are available from the “Supplementary data” link in the online posting of the article and from the same link in the online table of contents at https://academic.oup.com/cdn/.

JX, EKH, and ME contributed equally to this work.

Abbreviations used: CC, Collaborative Cross; ESI, electrospray ionization; HILIC/UPLC-MS/MS, hydrophilic interaction liquid chromatography ultra-performance liquid chromatography-tandem mass spectrometry; MSEA, Metabolite Set Enrichment Analyses; OPLS-DA, orthogonal partial least squares discriminant analysis; RP/UPLC-MS/MS, reverse phase ultra-performance liquid chromatography-tandem mass spectrometry; UNC, University of North Carolina; VDD, vitamin D deficient/deficiency; VDS, vitamin D sufficient; VIP, variable importance to projection; 25(OH)D, 25-hydroxyvitamin D.
