# Supplementary material for: Maternal Liver Metabolic Response to Chronic Vitamin D Deficiency Is Determined by Mouse Strain Genetic Background
Source: Curr Dev Nutr. 2020 Jun 20;4(8):nzaa106. doi: 10.1093/cdn/nzaa106 (PMC7439094; doi:10.1093/cdn/nzaa106)
Supplement: nzaa106_Supplemental_File [file nzaa106_supplemental_file.pdf]

Title: Maternal liver metabolic response to chronic vitamin D deficiency is determined by mouse strain genetic background

First author: Jing Xue

Online Supplementary Material

Supplemental Table 1a. Metabolites that make up the VDS-CC011 signature.

SUPER.PATHWAY	SUB.PATHWAY	Metabolite Name (Metabolon)	Metaboanalyst Match	VDS-CC011 vs. Rest VIP	VDS-CC011 vs. Rest VIP[1]cvSE	VDS-CC011 vs. Rest Fold Difference	VDS-CC011 vs. Rest Fold Difference Direction ("+"increased in VDD; "-"decreased)
Amino Acid	Alanine and Aspartate Metabolism	alanine	L-Alanine	1.69	1.4	1.4	+
Amino Acid	Glutamate Metabolism	N-acetylglutamate	N-Acetylglutamic acid	2.34	0.6	7.2	+
Amino Acid	Glutamate Metabolism	glutamine	L-Glutamine	1.79	0.4	2.0	-
Amino Acid	Glutamate Metabolism	glutamate	L-Glutamic acid	1.51	0.8	1.5	+
Amino Acid	Glycine, Serine and Threonine Metabolism	N-acetylserine	N-Acetylserine	1.65	0.8	2.2	+
Amino Acid	Glycine, Serine and Threonine Metabolism	N-acetylthreonine	NA	1.53	0.5	1.6	+
Amino Acid	Guanidino and Acetamido Metabolism	4-guanidinobutanoate	4-Guanidinobutanoic acid	1.68	0.8	4.7	+
Amino Acid	Histidine Metabolism	formiminoglutamate	Formiminoglutamic acid	2.09	1.0	4.7	+
Amino Acid	Histidine Metabolism	histidine	L-Histidine	1.92	0.9	2.4	+
Amino Acid	Leucine, Isoleucine and Valine Metabolism	isovalerylcarnitine	Isovalerylcarnitine	2.15	1.1	10.8	+
Amino Acid	Leucine, Isoleucine and Valine Metabolism	tiglylcarnitine	Tiglylcarnitine	1.73	1.5	4.7	+
Amino Acid	Leucine, Isoleucine and Valine Metabolism	2-methylbutyrylcarnitine (C5)	2-Methylbutyroylcarnitine	1.87	1.3	4.4	+
Amino Acid	Leucine, Isoleucine and Valine Metabolism	isobutyrylcarnitine	Isobutyryl-L-carnitine	1.68	1.4	3.3	+
Amino Acid	Leucine, Isoleucine and Valine Metabolism	beta-hydroxyisovalerate	3-Hydroxyisovaleric acid	1.73	1.3	2.1	+
Amino Acid	Leucine, Isoleucine and Valine Metabolism	3-hydroxyisobutyrate	(S)-3-Hydroxyisobutyric acid	1.47	1.0	1.9	+
Amino Acid	Leucine, Isoleucine and Valine Metabolism	valine	L-Valine	1.65	1.2	1.3	+
Amino Acid	Lysine Metabolism	2-aminoadipate	Aminoadipic acid	1.76	1.9	5.1	+
Amino Acid	Lysine Metabolism	5-aminovalerate	5-Aminopentanoic acid	2.09	0.8	3.1	+
Amino Acid	Lysine Metabolism	glutarate (pentanedioate)	Glutaric acid	1.66	1.7	2.5	+
Amino Acid	Lysine Metabolism	5-hydroxylysine	5-Hydroxylysine	1.63	1.1	2.1	+
Amino Acid	Lysine Metabolism	N6,N6,N6-trimethyllysine	N6,N6,N6-Trimethyl-L-lysine	1.87	0.9	1.8	+
Amino Acid	Lysine Metabolism	N6-acetyllysine	N6-Acetyl-L-lysine	1.6	1.1	1.7	+
Amino Acid	Methionine, Cysteine, SAM and Taurine Metabolism	cystine	L-Cystine	1.56	0.8	2.8	+
Amino Acid	Methionine, Cysteine, SAM and Taurine Metabolism	methionine sulfoxide	Methionine sulfoxide	1.6	1.1	1.7	+
Amino Acid	Methionine, Cysteine, SAM and Taurine Metabolism	2-aminobutyrate	L-Alpha-aminobutyric acid	1.83	1.2	1.7	+
Amino Acid	Phenylalanine and Tyrosine Metabolism	2-hydroxyphenylacetate	Ortho-Hydroxyphenylacetic acid	1.93	1.5	3.3	+
Amino Acid	Phenylalanine and Tyrosine Metabolism	phenol sulfate	NA	1.77	1.4	3.3	+
Amino Acid	Phenylalanine and Tyrosine Metabolism	N-formylphenylalanine	NA	1.52	0.6	2.3	+
Amino Acid	Tryptophan Metabolism	anthranilate	2-Aminobenzoic acid	1.84	1.2	3.7	+
Amino Acid	Tryptophan Metabolism	kynurenine	L-Kynurenine	1.81	1.3	3.3	+
Amino Acid	Tryptophan Metabolism	picolinate	Picolinic acid	1.75	1.3	3.3	+
Amino Acid	Tryptophan Metabolism	kynurenate	Kynurenic acid	1.49	0.5	3.0	+
Amino Acid	Urea cycle; Arginine and Proline Metabolism	citrulline	Citrulline	1.46	0.5	1.8	+
Amino Acid	Urea cycle; Arginine and Proline Metabolism	proline	L-Proline	1.55	0.9	1.3	+
Carbohydrate	Fructose, Mannose and Galactose Metabolism	fructose	D-Fructose	1.53	0.4	3.2	-
Carbohydrate	Glycolysis, Gluconeogenesis, and Pyruvate Metabolism	pyruvate	Pyruvic acid	1.55	0.6	2.7	-
Carbohydrate	Glycolysis, Gluconeogenesis, and Pyruvate Metabolism	glucose	D-Glucose	1.55	0.6	2.2	-
Carbohydrate	Pentose Metabolism	ribose	D-Ribose	1.61	0.8	2.1	-
Cofactors and Vitamins	Nicotinate and Nicotinamide Metabolism	nicotinamide	Niacinamide	1.73	0.6	1.5	-
Lipid	Diacylglycerol	palmitoyl-docosahexaenoyl-glycerol (16:0/22:6) [1]*	DG(16:0/22:6(4Z,7Z,10Z,13Z,16Z,19Z)/0:0)	1.63	1.6	2.9	+
Lipid	Diacylglycerol	palmitoyl-docosahexaenoyl-glycerol (16:0/22:6) [2]*	#2	1.45	1.2	2.4	+
Lipid	Diacylglycerol	oleoyl-arachidonoyl-glycerol (18:1/20:4) [2]*	DG(18:1(9Z)/20:4(5Z,8Z,11Z,14Z)/0:0)	1.7	0.3	2.2	+
Lipid	Diacylglycerol	palmitoyl-arachidonoyl-glycerol (16:0/20:4) [2]*	#2	1.86	1.2	2.0	+
Lipid	Eicosanoid	6-keto prostaglandin F1alpha	6-Keto-prostaglandin F1a	1.57	1.1	2.7	+
Lipid	Endocannabinoid	N-stearoyltaurine	NA	1.54	1.2	3.0	+
Lipid	Endocannabinoid	oleoyl ethanolamide	N-Oleoylethanolamine	1.51	0.6	1.7	+
Lipid	Fatty Acid Metabolism(Acyl Carnitine)	acetylcarnitine	L-Acetylcarnitine	1.56	1.1	2.8	+
Lipid	Fatty Acid, Amino	2-aminooctanoate	DL-2-Aminooctanoic acid	1.59	0.2	2.5	+
Lipid	Fatty Acid, Dicarboxylate	2-hydroxyadipate	2-Hydroxyadipic acid	1.78	1.9	5.5	+
Lipid	Fatty Acid, Dicarboxylate	maleate	Maleic acid	1.63	1.1	1.9	+
Lipid	Fatty Acid, Dihydroxy	14,15-DiHETrE	14,15-DiHETrE	1.51	1.7	1.9	+
Lipid	Glycerolipid Metabolism	glycerophosphoglycerol	NA	1.45	1.1	2.5	+
Lipid	Phospholipid Metabolism	1-palmitoyl-2-oleoyl-GPE (16:0/18:1)	PE(16:0/18:1(9Z))	1.48	0.9	1.5	+
Lipid	Polyunsaturated Fatty Acid (n3 and n6)	docosapentaenoate (n6 DPA; 22:5n6)	Docosapentaenoic acid (22n-6)	1.87	1.5	6.3	+
Lipid	Polyunsaturated Fatty Acid (n3 and n6)	adrenate (22:4n6)	Adrenic acid	1.77	1.0	2.6	+
Lipid	Polyunsaturated Fatty Acid (n3 and n6)	dihomo-linolenate (20:3n3 or n6)	8,11,14 Eicosatrienoic Acid	1.78	0.7	2.3	+
Lipid	Polyunsaturated Fatty Acid (n3 and n6)	dihomo-linoleate (20:2n6)	Eicosadienoic acid	1.52	0.9	2.1	+
Lipid	Polyunsaturated Fatty Acid (n3 and n6)	docosahexaenoate (DHA; 22:6n3)	Docosahexaenoic acid	1.47	0.5	1.6	+
Lipid	Primary Bile Acid Metabolism	beta-muricholate	3a,6b,7b-Trihydroxy-5b-cholanoic acid	1.58	1.7	2.5	+
Lipid	Sphingolipid Metabolism	sphinganine	Sphinganine	1.71	1.3	2.6	+
Lipid	Sphingolipid Metabolism	N-palmitoyl-sphingosine (d18:1/16:0)	Ceramide (d18:1/16:0)	1.47	1.0	1.9	+
Lipid	Sphingolipid Metabolism	N-palmitoyl-sphinganine (d18:0/16:0)	Cer(d18:0/16:0)	1.51	1.0	1.7	+
Lipid	Sphingolipid Metabolism	sphingosine	Sphingosine	1.48	0.6	1.7	+
Lipid	Sterol	campesterol	Campesterol	1.47	0.4	2.4	-
Nucleotide	Purine Metabolism, (Hypo)Xanthine/Inosine containing	allantoic acid	Allantoic acid	1.63	0.5	3.9	-
Nucleotide	Purine Metabolism, (Hypo)Xanthine/Inosine containing	N1-methylinosine	1-Methylinosine	1.75	1.4	2.6	+
Nucleotide	Purine Metabolism, (Hypo)Xanthine/Inosine containing	2'-deoxyinosine	Deoxyinosine	1.8	1.6	2.0	+
Nucleotide	Purine Metabolism, Adenine containing	2'-deoxyadenosine 5'-monophosphate	Deoxyadenosine monophosphate	1.66	1.0	2.3	+
Nucleotide	Purine Metabolism, Guanine containing	2'-deoxyguanosine 3'-monophosphate	NA	1.53	1.4	2.7	+
Nucleotide	Purine Metabolism, Guanine containing	2'-deoxyguanosine 5'-monophosphate (dGMP)	2'-Deoxyguanosine 5'-monophosphate	1.71	0.9	2.5	+
Nucleotide	Purine Metabolism, Guanine containing	2'-deoxyguanosine	Deoxyguanosine	1.66	1.3	1.9	+
Nucleotide	Pyrimidine Metabolism, Cytidine containing	N4-acetylcytidine	N4-Acetylcytidine	1.47	1.2	2.7	+
Nucleotide	Pyrimidine Metabolism, Cytidine containing	2'-deoxycytidine 5'-monophosphate	dCMP	1.89	1.3	2.5	+
Nucleotide	Pyrimidine Metabolism, Cytidine containing	5-methyl-2'-deoxycytidine	5-Methyldeoxycytidine	1.77	0.6	2.0	+
Nucleotide	Pyrimidine Metabolism, Cytidine containing	2'-deoxycytidine	Deoxycytidine	1.52	0.8	1.8	+
Nucleotide	Pyrimidine Metabolism, Thymine containing	thymidine	Thymidine	1.61	1.1	2.4	+
Nucleotide	Pyrimidine Metabolism, Uracil containing	pseudouridine	Pseudouridine	1.8	1.4	2.4	+
Nucleotide	Pyrimidine Metabolism, Uracil containing	2'-deoxyuridine	Deoxyuridine	1.87	0.8	2.3	+
Peptide	Gamma-glutamyl Amino Acid	gamma-glutamylphenylalanine	gamma-Glutamylphenylalanine	1.55	0.5	2.4	+
Peptide	Gamma-glutamyl Amino Acid	gamma-glutamyl-2-aminobutyrate	NA	1.87	0.3	2.3	+

Supplemental Table 2a. Metabolites that make up the VDD-CC011 signature.

SUPER.PATHWAY	SUB.PATHWAY	Metabolite Name (Metabolon)	Metaboanalyst Match	VDD-CC011 vs. Rest VIP	VDD-CC011 vs. Rest VIP[1]cvSE	VDD-CC011 vs. Rest Fold Difference	VDD-CC011 vs. Rest Fold Difference Direction ("+"increased in VDD; "-"decreased)
Amino Acid	Alanine and Aspartate Metabolism	aspartate	L-Aspartic acid	1.6	0.6	1.4	+
Amino Acid	Alanine and Aspartate Metabolism	alanine	L-Alanine	1.59	0.7	1.4	+
Amino Acid	Glutamate Metabolism	N-acetylglutamate	N-Acetylglutamic acid	2.1	0.8	6.8	+
Amino Acid	Glutamate Metabolism	glutamine	L-Glutamine	1.56	0.8	1.8	-
Amino Acid	Glycine, Serine and Threonine Metabolism	N-acetylserine	N-Acetylserine	1.77	0.7	2.3	+
Amino Acid	Glycine, Serine and Threonine Metabolism	N-acetylthreonine	NA	1.79	0.3	1.8	+
Amino Acid	Glycine, Serine and Threonine Metabolism	serine	L-Serine	1.79	0.9	1.4	+
Amino Acid	Glycine, Serine and Threonine Metabolism	threonine	L-Threonine	1.73	0.7	1.4	+
Amino Acid	Glycine, Serine and Threonine Metabolism	glycine	Glycine	1.57	0.7	1.4	+
Amino Acid	Guanidino and Acetamido Metabolism	4-guanidinobutanoate	4-Guanidinobutanoic acid	1.75	1.5	4.7	+
Amino Acid	Histidine Metabolism	imidazole propionate	Imidazolepropionic acid	1.48	0.9	3.2	+
Amino Acid	Histidine Metabolism	formiminoglutamate	Formiminoglutamic acid	1.53	1.5	2.8	+
Amino Acid	Histidine Metabolism	histidine	L-Histidine	1.66	0.4	2.0	+
Amino Acid	Leucine, Isoleucine and Valine Metabolism	isovalerylcarnitine	Isovalerylcarnitine	2.02	0.9	5.9	+
Amino Acid	Leucine, Isoleucine and Valine Metabolism	isovalerylglycine	Isovalerylglycine	1.89	1.1	3.8	+
Amino Acid	Leucine, Isoleucine and Valine Metabolism	2-methylbutyrylcarnitine (C5)	2-Methylbutyroylcarnitine	1.77	0.8	3.2	+
Amino Acid	Leucine, Isoleucine and Valine Metabolism	tiglylcarnitine	Tiglylcarnitine	1.63	1.1	3.1	+
Amino Acid	Leucine, Isoleucine and Valine Metabolism	3-methyl-2-oxobutyrate	Alpha-ketoisovaleric acid	1.53	1.2	3.0	+
Amino Acid	Leucine, Isoleucine and Valine Metabolism	beta-hydroxyisovalerate	3-Hydroxyisovaleric acid	1.58	1.4	2.0	+
Amino Acid	Leucine, Isoleucine and Valine Metabolism	valine	L-Valine	1.72	0.5	1.4	+
Amino Acid	Leucine, Isoleucine and Valine Metabolism	isoleucine	L-Isoleucine	1.57	0.9	1.3	+
Amino Acid	Lysine Metabolism	pipecolate	Pipecolic acid	1.81	1.0	2.7	+
Amino Acid	Lysine Metabolism	N6,N6,N6-trimethyllysine	N6,N6,N6-Trimethyl-L-lysine	1.62	0.4	1.6	+
Amino Acid	Methionine, Cysteine, SAM and Taurine Metabolism	2-aminobutyrate	L-Alpha-aminobutyric acid	1.79	0.7	1.6	+
Amino Acid	Methionine, Cysteine, SAM and Taurine Metabolism	methionine sulfoxide	Methionine sulfoxide	1.54	0.8	1.5	+
Amino Acid	Methionine, Cysteine, SAM and Taurine Metabolism	methionine	L-Methionine	1.81	0.9	1.4	+
Amino Acid	Phenylalanine and Tyrosine Metabolism	2-hydroxyphenylacetate	Ortho-Hydroxyphenylacetic acid	1.96	1.0	3.5	+
Amino Acid	Phenylalanine and Tyrosine Metabolism	phenylalanine	L-Phenylalanine	1.47	0.8	1.3	+
Amino Acid	Tryptophan Metabolism	anthranilate	2-Aminobenzoic acid	1.46	2.5	4.6	+
Amino Acid	Tryptophan Metabolism	picolinate	Picolinic acid	1.58	1.8	3.0	+
Amino Acid	Tryptophan Metabolism	kynurenine	L-Kynurenine	1.55	2.2	2.6	+
Amino Acid	Tryptophan Metabolism	tryptophan	L-Tryptophan	1.61	1.5	1.4	+
Amino Acid	Urea cycle; Arginine and Proline Metabolism	urea	Urea	1.69	0.7	1.8	+
Amino Acid	Urea cycle; Arginine and Proline Metabolism	proline	L-Proline	1.56	0.8	1.3	+
Amino Acid	Urea cycle; Arginine and Proline Metabolism	ornithine	Ornithine	1.6	0.8	1.3	+
Cofactors and Vitamins	Nicotinate and Nicotinamide Metabolism	nicotinamide	Niacinamide	1.64	0.5	1.4	-
Cofactors and Vitamins	Pantothenate and CoA Metabolism	pantothenate	Pantothenic acid	1.52	0.9	1.3	+
Lipid	Diacylglycerol	palmitoyl-arachidonoyl-glycerol (16:0/20:4) [2]*	#2	1.78	1.5	2.1	+
Lipid	Eicosanoid	6-keto prostaglandin F1alpha	6-Keto-prostaglandin F1a	1.84	1.6	5.7	+
Lipid	Eicosanoid	12-HHTrE	12S-HHT	1.91	1.0	4.1	+
Lipid	Eicosanoid	prostaglandin F2alpha	Prostaglandin F2a	1.67	0.9	2.8	+
Lipid	Fatty Acid, Dihydroxy	19,20-DiHDPA	19,20-DiHDPA	1.53	1.4	1.8	+
Lipid	Fatty Acid, Dihydroxy	14,15-DiHETrE	14,15-DiHETrE	1.54	1.1	1.8	+
Lipid	Lysolipid	1-palmitoyl-GPS (16:0)*	NA	1.54	2.3	5.3	+
Lipid	Polyunsaturated Fatty Acid (n3 and n6)	docosapentaenoate (n6 DPA; 22:5n6)	Docosapentaenoic acid (22n-6)	1.92	1.7	13.0	+
Lipid	Polyunsaturated Fatty Acid (n3 and n6)	adrenate (22:4n6)	Adrenic acid	1.88	0.8	2.9	+
Lipid	Polyunsaturated Fatty Acid (n3 and n6)	dihomo-linolenate (20:3n3 or n6)	8,11,14 Eicosatrienoic Acid	1.67	1.3	2.6	+
Lipid	Polyunsaturated Fatty Acid (n3 and n6)	docosahexaenoate (DHA; 22:6n3)	Docosahexaenoic acid	1.47	2.0	1.9	+
Lipid	Sphingolipid Metabolism	sphingomyelin (d18:1/18:1, d18:2/18:0)	NA	1.49	0.5	3.2	-
Lipid	Sphingolipid Metabolism	tricosanoyl sphingomyelin (d18:1/23:0)*	SM(d18:1/23:0)	1.54	0.4	2.5	-
Lipid	Sphingolipid Metabolism	lignoceroyl sphingomyelin (d18:1/24:0)	NA	1.47	0.5	2.2	-
Lipid	Sphingolipid Metabolism	sphingomyelin (d18:1/15:0, d16:1/17:0)*	NA	1.8	0.6	2.2	-
Lipid	Sphingolipid Metabolism	sphingomyelin (d18:1/17:0, d17:1/18:0, d19:1/16:0)	NA	1.79	0.7	2.2	-
Lipid	Sphingolipid Metabolism	N-palmitoyl-sphinganine (d18:0/16:0)	Cer(d18:0/16:0)	1.57	0.8	1.9	+
Lipid	Sphingolipid Metabolism	N-palmitoyl-sphingosine (d18:1/16:0)	Ceramide (d18:1/16:0)	1.86	1.1	1.8	+
Lipid	Sterol	campesterol	Campesterol	1.47	0.5	2.4	-
Nucleotide	Purine Metabolism, (Hypo)Xanthine/Inosine containing	N1-methylinosine	1-Methylinosine	1.74	0.8	2.4	+
Nucleotide	Purine Metabolism, Adenine containing	2'-deoxyadenosine 5'-monophosphate	Deoxyadenosine monophosphate	1.47	1.1	2.4	+
Nucleotide	Purine Metabolism, Guanine containing	2'-deoxyguanosine 5'-monophosphate (dGMP)	2'-Deoxyguanosine 5'-monophosphate	1.82	1.0	2.9	+
Nucleotide	Purine Metabolism, Guanine containing	7-methylguanine	7-Methylguanine	1.54	0.6	1.4	+
Nucleotide	Pyrimidine Metabolism, Cytidine containing	2'-deoxycytidine 5'-monophosphate	dCMP	1.73	1.1	2.1	+
Nucleotide	Pyrimidine Metabolism, Cytidine containing	5-methyl-2'-deoxycytidine	5-Methyldeoxycytidine	1.55	1.1	2.0	+
Nucleotide	Pyrimidine Metabolism, Thymine containing	thymidine	Thymidine	1.85	1.1	2.7	+
Nucleotide	Pyrimidine Metabolism, Uracil containing	uridine 5'-monophosphate (UMP)	Uridine 5'-monophosphate	1.46	1.6	2.6	+
Nucleotide	Pyrimidine Metabolism, Uracil containing	2'-deoxyuridine	Deoxyuridine	1.65	1.8	2.5	+
Nucleotide	Pyrimidine Metabolism, Uracil containing	pseudouridine	Pseudouridine	1.78	0.1	2.1	+
Peptide	Acetylated Peptides	phenylacetylglycine	Phenylacetylglycine	1.71	1.3	2.6	+
Peptide	Gamma-glutamyl Amino Acid	gamma-glutamyl-2-aminobutyrate	NA	1.64	0.3	2.3	+
Peptide	Gamma-glutamyl Amino Acid	gamma-glutamyl-epsilon-lysine	Epsilon-(gamma-Glutamyl)-lysine	1.79	0.9	1.9	+
Xenobiotics	Chemical	O-sulfo-L-tyrosine	NA	1.48	0.5	1.6	+

Supplemental Table 3a. Metabolites that make up the VDS-CC042 signature.

SUPER.PATHWAY	SUB.PATHWAY	Metabolite Name (Metabolon)	Metaboanalyst Match	VDS-CC042 vs. Rest VIP	VDS-CC042 vs. Rest VIP[1]cvSE	VDS-CC042 vs. Rest Fold Difference	Fold Difference Direction ("+"increased in VDD; "-"decreased)
Amino Acid	Alanine and Aspartate Metabolism	N-acetylasparagine	N-Acetylasparagine	2.2	1.4	2.4	+
Amino Acid	Alanine and Aspartate Metabolism	aspartate	L-Aspartic acid	1.9	0.9	1.7	-
Amino Acid	Glutamate Metabolism	N-acetylglutamine	N-Acetylglutamine	1.9	0.7	2.3	+
Amino Acid	Glycine, Serine and Threonine Metabolism	glycine	Glycine	1.5	1.0	1.2	-
Amino Acid	Histidine Metabolism	imidazole propionate	Imidazolepropionic acid	1.6	1.9	2.9	+
Amino Acid	Methionine, Cysteine, SAM and Taurine Metabolism	S-adenosylmethionine (SAM)	S-Adenosylmethionine	1.7	1.3	3.3	+
Amino Acid	Phenylalanine and Tyrosine Metabolism	p-cresol sulfate	p-Cresol sulfate	1.5	0.5	16.4	-
Amino Acid	Phenylalanine and Tyrosine Metabolism	4-hydroxyphenylpyruvate	4-Hydroxyphenylpyruvic acid	1.8	1.3	2.8	+
Amino Acid	Phenylalanine and Tyrosine Metabolism	tyrosine	L-Tyrosine	1.6	0.7	1.9	-
Amino Acid	Polyamine Metabolism	5-methylthioadenosine (MTA)	5'-Methylthioadenosine	1.8	1.1	2.3	+
Amino Acid	Urea cycle; Arginine and Proline Metabolism	citrulline	Citrulline	1.6	0.4	2.5	-
Amino Acid	Urea cycle; Arginine and Proline Metabolism	ornithine	Ornithine	1.6	0.8	1.3	-
Amino Acid	Urea cycle; Arginine and Proline Metabolism	proline	L-Proline	1.5	0.7	1.3	-
Carbohydrate	Aminosugar Metabolism	N-acetylglucosamine 6-phosphate	N-Acetylglucosamine 6-phosphate	2.0	1.6	2.9	+
Carbohydrate	Fructose, Mannose and Galactose Metabolism	2-ketogulonate	2-Keto-L-gluconate	1.6	1.6	2.1	+
Carbohydrate	Nucleotide Sugar	UDP-N-acetylglucosamine/galactosamine	Uridine diphosphate-N-acetylglucosamine/Uridine diphosphate-N-acetylgalactosamine	2.4	1.4	3.8	+
Carbohydrate	Pentose Metabolism	ribitol	Ribitol	1.7	0.6	5.6	-
Carbohydrate	Pentose Metabolism	arabonate/xylonate	NA	1.7	1.8	1.6	+
Carbohydrate	Pentose Metabolism	ribose	D-Ribose	1.5	0.7	1.5	+
Carbohydrate	Pentose Phosphate Pathway	ribose 1-phosphate	Ribose 1-phosphate	1.6	0.7	1.5	+
Cofactors and Vitamins	Ascorbate and Aldarate Metabolism	gulonate*	Gulonic acid	1.5	1.7	1.5	+
Cofactors and Vitamins	Pantothenate and CoA Metabolism	phosphopantetheine	Pantetheine 4'-phosphate	1.6	2.3	4.8	+
Cofactors and Vitamins	Pantothenate and CoA Metabolism	coenzyme A	Coenzyme A	1.5	1.4	3.0	+
Cofactors and Vitamins	Pantothenate and CoA Metabolism	pantothenate	Pantothenic acid	1.5	0.5	1.3	-
Energy	TCA Cycle	citrate	Citric acid	1.7	1.0	3.7	+
Energy	TCA Cycle	aconitate [cis or trans]	Aconitic Acid	1.6	1.0	3.4	+
Lipid	Endocannabinoid	oleoyl ethanolamide	N-Oleoylethanolamine	1.5	0.8	2.0	-
Lipid	Fatty Acid Metabolism(Acyl Carnitine)	palmitoleoylcarnitine*	NA	1.5	1.0	1.8	+
Lipid	Fatty Acid, Monohydroxy	3-hydroxyoctanoate	3-Hydroxyoctanoic acid	1.7	1.1	3.1	+
Lipid	Fatty Acid, Monohydroxy	4-hydroxybutyrate (GHB)	4-Hydroxybutyric acid	1.9	2.1	2.9	+
Lipid	Lysolipid	1-linoleoyl-GPI (18:2)*	NA	1.6	1.9	3.6	+
Lipid	Lysolipid	1-linolenoyl-GPC (18:3)*	LysoPC(18:3(9Z,12Z,15Z))	1.8	2.1	2.7	+
Lipid	Lysolipid	1-stearoyl-GPE (18:0)	LysoPE(18:0/0:0)	1.9	0.9	1.6	-
Lipid	Lysolipid	1-palmitoyl-GPE (16:0)	LysoPE(16:0/0:0)	1.6	0.8	1.3	-
Lipid	Phospholipid Metabolism	1-stearoyl-2-oleoyl-GPI (18:0/18:1)*	NA	1.6	2.1	3.5	+
Lipid	Phospholipid Metabolism	1-stearoyl-2-linoleoyl-GPI (18:0/18:2)	NA	2.0	2.4	3.1	+
Lipid	Sphingolipid Metabolism	phytosphingosine	Phytosphingosine	2.1	1.6	2.7	+
Lipid	Sphingolipid Metabolism	N-margaroyl-sphingosine (d18:1/17:0)*	NA	1.7	0.5	2.6	-
Lipid	Sphingolipid Metabolism	N-stearoyl-sphingosine (d18:1/18:0)*	Ceramide (d18:1/18:0)	1.5	0.6	2.3	-
Lipid	Sphingolipid Metabolism	sphingomyelin (d18:1/17:0, d17:1/18:0, d19:1/16:0)	NA	1.5	0.3	1.9	-
Nucleotide	Purine Metabolism, (Hypo)Xanthine/Inosine containing	inosine	Inosine	2.0	0.8	2.1	+
Nucleotide	Purine Metabolism, Adenine containing	adenylosuccinate	Adenylsuccinic acid	2.2	1.4	10.4	+
Nucleotide	Purine Metabolism, Adenine containing	adenosine 3',5'-diphosphate	Adenosine 3',5'-diphosphate	2.0	1.1	2.5	+
Nucleotide	Purine Metabolism, Adenine containing	N6-succinyladenosine	Succinyladenosine	1.5	0.8	2.0	+
Nucleotide	Purine Metabolism, Guanine containing	N2,N2-dimethylguanosine	N2,N2-Dimethylguanosine	1.5	0.8	2.1	-
Nucleotide	Pyrimidine Metabolism, Uracil containing	uridine 5'-monophosphate (UMP)	Uridine 5'-monophosphate	1.7	2.7	4.0	+
Nucleotide	Pyrimidine Metabolism, Uracil containing	2'-deoxyuridine	Deoxyuridine	1.7	0.3	3.4	-
Peptide	Dipeptide	valylleucine	Valyl-Leucine	2.2	1.9	5.5	+
Peptide	Dipeptide	leucylglutamine*	Leucyl-Glutamine	1.8	1.9	4.7	+
Peptide	Dipeptide	valylglutamine	Valyl-Glutamine	1.9	2.0	4.3	+
Peptide	Dipeptide	alanylleucine	Alanyl-Leucine	1.9	1.7	4.3	+
Peptide	Dipeptide	valylglycine	Valyl-Glycine	2.0	1.4	4.0	+
Peptide	Dipeptide	isoleucylglycine	Isoleucyl-Glycine	1.9	1.5	3.2	+
Peptide	Dipeptide	leucylglycine	Glycylleucine	1.7	1.2	3.0	+
Peptide	Dipeptide	phenylalanylglycine	Phenylalanyl-Glycine	1.8	1.8	2.9	+
Peptide	Dipeptide	tyrosylglycine	Tyrosyl-Glycine	1.8	1.0	2.8	+
Peptide	Dipeptide	glycylleucine	Glycylleucine	1.9	1.3	2.7	+
Peptide	Dipeptide	phenylalanylalanine	Phenylalanyl-Alanine	1.6	1.7	2.6	+
Peptide	Dipeptide	glycylvaline	Glycyl-Valine	1.8	1.0	2.3	+
Peptide	Gamma-glutamyl Amino Acid	gamma-glutamylthreonine	gamma-Glutamylthreonine	1.7	0.6	5.2	-
Peptide	Gamma-glutamyl Amino Acid	gamma-glutamylisoleucine*	gamma-Glutamylisoleucine	1.8	1.0	2.3	-
Peptide	Gamma-glutamyl Amino Acid	gamma-glutamylvaline	gamma-Glutamylvaline	1.6	0.8	2.2	-
Xenobiotics	Food Component/Plant	erythritol	Erythritol	1.5	0.6	4.2	-

Supplemental Table 4a. Metabolites that make up the VDD-CC017 signature.

SUPER.PATHWAY	SUB.PATHWAY	Metabolite Name (Metabolon)	Metaboanalyst Match	VDD-CC017 vs. Rest VIP	VDD-CC017 vs. Rest VIP[1]cvSE	VDD-CC017 vs. Rest Fold Difference	VDD-CC017 vs. Rest Fold Difference Direction ("+"increased in VDD; "-"decreased)
Amino Acid	Alanine and Aspartate Metabolism	N-acetylaspartate (NAA)	N-Acetyl-L-aspartate	2.1	0.5	3.5	+
Amino Acid	Glutathione Metabolism	glutathione, reduced (GSH)	Glutathione	1.6	1.6	5.0	+
Amino Acid	Glutathione Metabolism	cysteinylglycine	Cysteinylglycine	2.0	0.8	4.7	+
Amino Acid	Histidine Metabolism	1-methylhistidine	1-Methylhistidine	1.6	0.8	1.8	-
Amino Acid	Lysine Metabolism	5-(galactosylhydroxy)-L-lysine	Galactosylhydroxylysine	1.8	0.7	1.5	+
Amino Acid	Methionine, Cysteine, SAM and Taurine Metabolism	S-methylmethionine	NA	1.7	1.6	78.9	+
Amino Acid	Methionine, Cysteine, SAM and Taurine Metabolism	cysteine	L-Cysteine	2.1	0.8	3.8	+
Amino Acid	Methionine, Cysteine, SAM and Taurine Metabolism	cystathionine	L-Cystathionine	2.0	0.6	2.1	+
Amino Acid	Methionine, Cysteine, SAM and Taurine Metabolism	hypotaurine	Hypotaurine	1.9	1.0	2.0	+
Amino Acid	Methionine, Cysteine, SAM and Taurine Metabolism	N-acetyltaurine	NA	1.5	0.6	1.8	-
Amino Acid	Polyamine Metabolism	Spermidine	Spermidine	1.6	1.0	1.6	-
Amino Acid	Tryptophan Metabolism	thioproline	NA	2.0	1.0	3.8	+
Amino Acid	Urea cycle; Arginine and Proline Metabolism	arginine	L-Arginine	1.5	1.5	1.6	+
Carbohydrate	Fructose, Mannose and Galactose Metabolism	fructose	D-Fructose	1.5	0.8	1.9	+
Carbohydrate	Glycogen Metabolism	maltopentaose	Maltopentaose	1.5	1.1	2.7	+
Carbohydrate	Pentose Metabolism	ribulose/xylulose	D-Ribulose	2.0	1.1	1.8	+
Carbohydrate	Pentose Metabolism	ribose	D-Ribose	1.7	0.6	1.6	+
Cofactors and Vitamins	Ascorbate and Aldarate Metabolism	oxalate (ethanedioate)	Oxalic acid	1.5	0.8	2.0	-
Cofactors and Vitamins	Nicotinate and Nicotinamide Metabolism	trigonelline (N'-methylnicotinate)	Trigonelline	1.9	1.6	4.6	+
Cofactors and Vitamins	Nicotinate and Nicotinamide Metabolism	nicotinamide riboside	Nicotinamide riboside	1.7	1.1	2.6	+
Cofactors and Vitamins	Nicotinate and Nicotinamide Metabolism	1-methylnicotinamide	1-Methylnicotinamide	1.6	1.5	2.0	+
Cofactors and Vitamins	Pantothenate and CoA Metabolism	coenzyme A	Coenzyme A	1.7	1.6	4.9	+
Cofactors and Vitamins	Pantothenate and CoA Metabolism	3'-dephosphocoenzyme A	Dephospho-CoA	1.8	1.3	4.9	+
Cofactors and Vitamins	Pantothenate and CoA Metabolism	phosphopantetheine	Pantetheine 4'-phosphate	1.5	1.5	4.3	+
Cofactors and Vitamins	Tocopherol Metabolism	gamma-tocopherol/beta-tocopherol	NA	1.6	2.0	3.6	+
Cofactors and Vitamins	Tocopherol Metabolism	alpha-tocopherol	Alpha-Tocopherol	1.8	1.3	2.3	+
Lipid	Fatty Acid Metabolism (also BCAA Metabolism)	methylmalonate (MMA)	Methylmalonic acid	1.6	1.4	1.7	+
Lipid	Fatty Acid Metabolism(Acyl Carnitine)	stearoylcarnitine	Stearoylcarnitine	1.5	1.5	2.1	+
Lipid	Fatty Acid Metabolism(Acyl Glycine)	hexanoylglycine	Hexanoylglycine	1.6	1.7	2.1	+
Lipid	Fatty Acid Synthesis	malonylcarnitine	Malonylcarnitine	1.5	0.8	1.8	+
Lipid	Fatty Acid, Dicarboxylate	hexadecanedioate	Hexadecanedioic acid	1.7	0.8	1.8	+
Lipid	Fatty Acid, Monohydroxy	3-hydroxylaurate	3-Hydroxydodecanoic acid	1.7	1.1	1.8	+
Lipid	Fatty Acid, Monohydroxy	3-hydroxydecanoate	3-Hydroxycapric acid	1.6	0.6	1.7	+
Lipid	Glycerolipid Metabolism	glycerol	Glycerol	1.5	1.7	2.2	+
Lipid	Inositol Metabolism	inositol 1-phosphate (I1P)	Myo-inositol 1-phosphate	1.6	1.2	2.3	+
Lipid	Long Chain Fatty Acid	10-nonadecenoate (19:1n9)	Nonadeca-10(Z)-enoic acid	1.8	1.0	3.0	+
Lipid	Long Chain Fatty Acid	eicosenoate (20:1)	Eicosenoic acid	1.7	1.2	2.9	+
Lipid	Long Chain Fatty Acid	10-heptadecenoate (17:1n7)	NA	1.7	0.8	2.5	+
Lipid	Long Chain Fatty Acid	margarate (17:0)	Heptadecanoic acid	1.7	0.7	2.2	+
Lipid	Long Chain Fatty Acid	pentadecanoate (15:0)	Pentadecanoic acid	1.6	0.6	1.8	+
Lipid	Long Chain Fatty Acid	oleate/vaccenate (18:1)	Oleic acid	1.6	0.8	1.6	+
Lipid	Lysoplasmalogen	1-(1-enyl-oleoyl)-2-linoleoyl-GPE (P-18:1/18:2)*	NA	1.7	1.1	1.8	+
Lipid	Monoacylglycerol	1-linoleoylglycerol (18:2)	MG(18:2(9Z,12Z)/0:0/0:0)	1.5	1.8	3.5	+
Lipid	Monoacylglycerol	2-linoleoylglycerol (18:2)	MG(0:0/18:2(9Z,12Z)/0:0)	1.4	1.6	3.1	+
Lipid	Monoacylglycerol	1-oleoylglycerol (18:1)	MG(18:1(9Z)/0:0/0:0)	1.5	1.4	2.9	+
Lipid	Phosphatidylserine (PS)	1-stearoyl-2-arachidonoyl-GPS (18:0/20:4)	NA	1.5	0.7	1.6	-
Lipid	Phospholipid Metabolism	glycerophosphoethanolamine	Glycerylphosphorylethanolamine	1.7	1.5	2.4	+
Lipid	Phospholipid Metabolism	glycerophosphorylcholine (GPC)	Glycerophosphocholine	1.9	1.1	2.0	+
Lipid	Plasmalogen	1-(1-enyl-palmitoyl)-2-oleoyl-GPE (P-16:0/18:1)*	PE(16:0/18:1(9Z))	1.8	1.2	1.6	+
Lipid	Sterol	beta-sitosterol	Beta-Sitosterol	1.6	1.1	1.9	+
Nucleotide	Purine Metabolism, (Hypo)Xanthine/Inosine containing	2'-deoxyinosine	Deoxyinosine	1.6	0.9	1.7	+
Nucleotide	Purine Metabolism, (Hypo)Xanthine/Inosine containing	allantoin	Allantoin	1.5	0.4	1.6	-
Nucleotide	Purine Metabolism, (Hypo)Xanthine/Inosine containing	hypoxanthine	Hypoxanthine	1.8	0.9	1.4	+
Nucleotide	Purine Metabolism, Guanine containing	guanosine 3'-monophosphate (3'-GMP)	Guanosine 3'-phosphate	1.5	1.3	1.8	+
Nucleotide	Purine Metabolism, Guanine containing	guanine	Guanine	1.7	0.9	1.8	+
Nucleotide	Pyrimidine Metabolism, Cytidine containing	cytidine 3'-monophosphate (3'-CMP)	Cytidine monophosphate (all ' equivalent)	1.5	2.2	4.0	+
Nucleotide	Pyrimidine Metabolism, Orotate containing	orotidine	Orotidine	2.4	0.7	6.1	+
Nucleotide	Pyrimidine Metabolism, Uracil containing	uridine 3'-monophosphate (3'-UMP)	NA	1.6	1.5	1.7	+
Peptide	Dipeptide	alanylleucine	Alanyl-Leucine	2.0	0.5	4.7	+
Peptide	Dipeptide	leucylglutamine*	Leucyl-Glutamine	1.6	0.7	3.4	+
Peptide	Dipeptide	valylglutamine	Valyl-Glutamine	1.8	0.7	3.4	+
Peptide	Dipeptide	valylleucine	Valyl-Leucine	1.5	0.7	3.3	+
Peptide	Dipeptide	phenylalanylalanine	Phenylalanyl-Alanine	1.5	0.5	2.2	+
Peptide	Gamma-glutamyl Amino Acid	gamma-glutamylcysteine	gamma-Glutamylcysteine	1.8	1.0	5.2	+
Xenobiotics	Bacterial/Fungal	tartronate (hydroxymalonate)	Hydroxypropanedioic acid	1.6	0.8	2.5	-
Xenobiotics	Drug	S-carboxymethyl-L-cysteine	S-Carboxymethyl-L-cysteine	1.5	1.2	2.3	+

Supplemental Table 5a. Metabolites that make up the VDD signature after adjustment for strain.

SUPER.PATHWAY	SUB.PATHWAY	Metabolite Name (Metabolon)	Metaboanalyst Match	VDS vs. VDD rmsVIP	VDS vs. VDD OrthVIP	VDS vs. VDD PredVIP	VDS vs. VDD Fold Difference
Amino Acid	Glutathione Metabolism	4-hydroxy-nonenal-glutathione	4-Hydroxynonenal	1.7	1.9	1.4	-1.6
Amino Acid	Glutathione Metabolism	ophthalmate	Ophthalmic acid	1.6	0.1	2.3	1.7
Amino Acid	Leucine, Isoleucine and Valine Metabolism	alpha-hydroxyisovalerate	2-Hydroxy-3-methylbutyric acid	1.7	1.3	2.0	-1.2
Amino Acid	Lysine Metabolism	pipecolate	Pipecolic acid	1.6	1.5	1.6	-1.3
Amino Acid	Methionine, Cysteine, SAM and Taurine Metabolism	2-aminobutyrate	L-Alpha-aminobutyric acid	1.7	1.5	1.8	1.1
Amino Acid	Methionine, Cysteine, SAM and Taurine Metabolism	S-adenosylmethionine (SAM)	S-Adenosylmethionine	1.6	0.9	2.1	1.8
Amino Acid	Phenylalanine and Tyrosine Metabolism	p-cresol sulfate	p-Cresol sulfate	2.6	0.9	3.5	2.9
Amino Acid	Phenylalanine and Tyrosine Metabolism	p-cresol-glucuronide*	p-Cresol glucuronide	2.1	0.9	2.8	2.2
Amino Acid	Polyamine Metabolism	5-methylthioadenosine (MTA)	5'-Methylthioadenosine	1.7	0.3	2.4	1.5
Amino Acid	Polyamine Metabolism	putrescine	Putrescine	1.5	0.8	1.9	-1.4
Amino Acid	Tryptophan Metabolism	indole-3-carboxylic acid	Indole-3-carboxylic acid	1.7	1.5	1.8	-1.2
Amino Acid	Urea cycle; Arginine and Proline Metabolism	argininosuccinate	Argininosuccinic acid	1.6	1.8	1.4	1.3
Amino Acid	Urea cycle; Arginine and Proline Metabolism	arginine	L-Arginine	1.9	1.9	1.9	1.4
Amino Acid	Urea cycle; Arginine and Proline Metabolism	homoarginine	Homo-L-arginine	1.7	0.9	2.2	-1.9
Carbohydrate	Aminosugar Metabolism	N-acetylneuraminate	N-Acetylneuraminic acid	1.8	1.8	1.7	-1.2
Cofactors and Vitamins	Nicotinate and Nicotinamide Metabolism	nicotinate ribonucleoside	Nicotinate D-ribonucleoside	1.7	0.9	2.2	-1.4
Lipid	Diacylglycerol	oleoyl-oleoyl-glycerol (18:1/18:1) [2]*	NA	2.3	2.2	2.4	1.4
Lipid	Diacylglycerol	oleoyl-linoleoyl-glycerol (18:1/18:2) [2]	NA	1.7	1.6	1.8	1.3
Lipid	Diacylglycerol	oleoyl-oleoyl-glycerol (18:1/18:1) [1]*	NA	2.0	2.2	1.7	1.7
Lipid	Endocannabinoid	oleoyl ethanolamide	N-Oleoylethanolamine	1.7	1.6	1.8	1.3
Lipid	Fatty Acid, Amino	2-aminoheptanoate	NA	1.5	1.8	1.0	-1.3
Lipid	Fatty Acid, Monohydroxy	4-hydroxybutyrate (GHB)	4-Hydroxybutyric acid	1.5	1.5	1.4	1.4
Lipid	Glycerolipid Metabolism	glycerophosphoglycerol	NA	1.8	1.7	1.9	1.5
Lipid	Long Chain Fatty Acid	eicosenoate (20:1)	Eicosenoic acid	1.9	2.5	1.1	1.7
Lipid	Long Chain Fatty Acid	palmitoleate (16:1n7)	Palmitoleic acid	1.8	2.4	0.6	1.4
Lipid	Long Chain Fatty Acid	10-nonadecenoate (19:1n9)	Nonadeca-10(Z)-enoic acid	1.9	2.5	1.1	1.7
Lipid	Long Chain Fatty Acid	10-heptadecenoate (17:1n7)	NA	2.0	2.2	1.7	1.7
Lipid	Long Chain Fatty Acid	oleate/vaccenate (18:1)	Oleic acid	2.3	2.8	1.6	1.4
Lipid	Long Chain Fatty Acid	palmitate (16:0)	Palmitic acid	1.5	2.1	0.4	1.2
Lipid	Long Chain Fatty Acid	myristate (14:0)	Myristic acid	1.5	2.1	0.4	1.2
Lipid	Long Chain Fatty Acid	pentadecanoate (15:0)	Pentadecanoic acid	1.6	2.2	0.6	1.2
Lipid	Long Chain Fatty Acid	stearate (18:0)	Stearic acid	1.5	2.0	0.4	1.2
Lipid	Long Chain Fatty Acid	margarate (17:0)	Heptadecanoic acid	1.6	2.2	0.6	1.3
Lipid	Lysolipid	1-palmitoleoyl-GPC (16:1)*	LysoPC(16:1(9Z))	1.8	1.1	2.3	1.4
Lipid	Lysolipid	1-oleoyl-GPC (18:1)	LysoPC(18:1(9Z))	2.1	1.4	2.7	1.4
Lipid	Lysolipid	1-oleoyl-GPE (18:1)	LysoPE(18:1(9Z)/0:0)	1.5	0.9	2.0	1.5
Lipid	Lysolipid	1-oleoyl-GPG (18:1)*	NA	2.0	1.6	2.4	2.3
Lipid	Lysolipid	1-linoleoyl-GPG (18:2)*	NA	1.5	1.4	1.7	1.5
Lipid	Monoacylglycerol	1-palmitoleoylglycerol (16:1)*	MG(16:1(9Z)/0:0/0:0)	1.6	2.2	0.3	1.5
Lipid	Monoacylglycerol	1-dihomo-linolenylglycerol (20:3)	MG(20:3(8Z,11Z,14Z)/0:0/0:0)	1.7	2.4	0.4	1.6
Lipid	Monoacylglycerol	1-arachidonylglycerol (20:4)	MG(20:4(5Z,8Z,11Z,14Z)/0:0/0:0)	1.6	2.2	0.3	1.4
Lipid	Monoacylglycerol	2-palmitoylglycerol (16:0)	MG(0:0/16:0/0:0)	1.6	2.1	0.9	1.1
Lipid	Monoacylglycerol	2-docosahexaenoylglycerol (22:6)*	MG(0:0/22:6(4Z,7Z,10Z,13Z,16Z,19Z)/0:0)	1.5	1.9	0.9	-1.0
Lipid	Monoacylglycerol	1-oleoylglycerol (18:1)	MG(18:1(9Z)/0:0/0:0)	1.7	2.3	0.3	1.8
Lipid	Monoacylglycerol	1-docosahexaenoylglycerol (22:6)	MG(22:6(4Z,7Z,10Z,13Z,16Z,19Z)/0:0/0:0)	1.5	1.9	0.9	1.1
Lipid	Monoacylglycerol	1-palmitoylglycerol (16:0)	MG(16:0/0:0/0:0)	1.5	1.9	0.8	1.2
Lipid	Monoacylglycerol	2-palmitoleoylglycerol (16:1)*	NA	1.6	2.2	0.4	1.4
Lipid	Monoacylglycerol	2-myristoylglycerol (14:0)	MG(0:0/14:0/0:0)	1.5	1.8	1.0	1.1
Lipid	Monoacylglycerol	2-oleoylglycerol (18:1)	MG(0:0/18:1(9Z)/0:0)	1.7	2.3	0.2	1.6
Lipid	Monoacylglycerol	1-linoleoylglycerol (18:2)	MG(18:2(9Z,12Z)/0:0/0:0)	1.5	2.1	0.3	1.6
Lipid	Phospholipid Metabolism	glycerophosphorylcholine (GPC)	Glycerophosphocholine	1.8	1.8	1.9	1.3
Lipid	Phospholipid Metabolism	glycerophosphoethanolamine	Glycerylphosphorylethanolamine	1.7	1.6	1.8	1.5
Lipid	Phospholipid Metabolism	1-stearoyl-2-oleoyl-GPE (18:0/18:1)	PE(18:0/18:1(9Z))	1.5	0.6	2.1	1.2
Lipid	Phospholipid Metabolism	1-oleoyl-2-linoleoyl-GPE (18:1/18:2)*	PE(18:1(9Z)/18:2(9Z,12Z))	1.6	0.7	2.1	1.3
Lipid	Phospholipid Metabolism	1-linoleoyl-2-arachidonoyl-GPE (18:2/20:4)*	NA	1.6	0.9	2.1	1.3
Lipid	Phospholipid Metabolism	1-stearoyl-2-arachidonoyl-GPE (18:0/20:4)	PE(18:0/20:4(5Z,8Z,11Z,14Z))	2.0	2.1	2.0	-1.0
Lipid	Phospholipid Metabolism	1-palmitoyl-2-arachidonoyl-GPE (16:0/20:4)*	PE(16:0/20:4(5Z,8Z,11Z,14Z))	2.0	2.3	1.7	-1.1
Lipid	Phospholipid Metabolism	1-palmitoyl-2-arachidonoyl-GPI (16:0/20:4)*	NA	1.9	1.7	2.0	-1.3
Lipid	Phospholipid Metabolism	1-palmitoyl-2-arachidonoyl-GPC (16:0/20:4n6)	PC(16:0/20:4(5Z,8Z,11Z,14Z))	1.8	1.5	2.0	-1.0
Lipid	Polyunsaturated Fatty Acid (n3 and n6)	linoleate (18:2n6)	Linoleic acid	1.5	2.0	0.7	1.3
Lipid	Polyunsaturated Fatty Acid (n3 and n6)	eicosapentaenoate (EPA; 20:5n3)	Eicosapentaenoic acid	1.5	1.8	1.0	1.4
Lipid	Polyunsaturated Fatty Acid (n3 and n6)	dihomo-linoleate (20:2n6)	Eicosadienoic acid	1.6	2.2	0.3	1.3
Lipid	Sphingolipid Metabolism	sphingomyelin (d18:1/14:0, d16:1/16:0)*	SM(d18:0/14:0)	1.8	0.1	2.5	-1.4
Lipid	Sphingolipid Metabolism	sphingomyelin (d18:1/15:0, d16:1/17:0)*	sphingomyelin	1.5	0.2	2.1	-1.2
Lipid	Steroid	corticosterone	Corticosterone	1.5	1.3	1.7	-2.0
Nucleotide	Purine Metabolism, (Hypo)Xanthine/Inosine containing	allantoic acid	Allantoic acid	1.5	0.3	2.2	1.3
Nucleotide	Purine Metabolism, Adenine containing	N6-methyladenosine	N6-Methyladenosine	1.5	1.0	1.9	-1.1
Nucleotide	Purine Metabolism, Guanine containing	guanine	Guanine	1.7	0.4	2.3	1.3
Nucleotide	Purine Metabolism, Guanine containing	7-methylguanine	7-Methylguanine	1.6	1.8	1.4	-1.1
Nucleotide	Purine Metabolism, Guanine containing	2'-deoxyguanosine 3'-monophosphate	deoxyguanosine	1.6	1.2	1.9	1.4
Nucleotide	Pyrimidine Metabolism, Cytidine containing	cytidine	Cytidine	1.9	1.2	2.4	-1.2
Peptide	Dipeptide	phenylalanylalanine	Phenylalanyl-Alanine	1.6	1.8	1.4	1.6
Peptide	Dipeptide	tyrosylglycine	Tyrosyl-Glycine	1.5	1.0	1.8	1.4
Peptide	Gamma-glutamyl Amino Acid	gamma-glutamyltyrosine	gamma-Glutamyltyrosine	1.5	2.1	0.3	1.4
Xenobiotics	Bacterial/Fungal	tartronate (hydroxymalonate)	Hydroxypropanedioic acid	1.5	2.1	0.4	-1.0
Xenobiotics	Chemical	sulfate*	Sulfate	1.6	1.6	1.7	-1.1
Xenobiotics	Drug	S-carboxymethyl-L-cysteine	S-Carboxymethyl-L-cysteine	1.5	0.7	1.9	2.0
Xenobiotics	Food Component/Plant	beta-guanidinopropanoate	Beta-Guanidinopropionic acid	2.1	0.3	3.0	2.2

Supplemental Table 1b. Results of Metabolite Set Enrichment Analyses (MSEA) for the VDS-CC011 signature.

**Metaboanalyst Pathway-SMHDP-Run 2/14/20**	**total**	**expected**	**hits**	**Raw p**	**Holm p**	**FDR**	**Metabolites in significantly enriched pathways**
Glucose-Alanine Cycle	13	0.901	4	**0.00941**	0.923	0.923	D-Glucose; L-Glutamic acid; L-Alanine; Pyruvic acid
Alpha Linolenic Acid and Linoleic Acid Metabolism	19	1.32	4	**0.0372**	1	1	Docosapentaenoic acid (22n-6); Docosahexaenoic acid; Adrenic acid; 8,11,14-Eicosatrienoic acid
Urea Cycle	29	2.01	5	**0.0446**	1	1	L-Glutamic acid; L-Alanine; Pyruvic acid; L-Glutamine; Citrulline
Alanine Metabolism	17	1.18	3	0.108	1	1	
Ammonia Recycling	32	2.22	4	0.176	1	1	
Amino Sugar Metabolism	33	2.29	4	0.19	1	1	
Methylhistidine Metabolism	4	0.277	1	0.25	1	1	
Pyrimidine Metabolism	59	4.09	5	0.39	1	1	
Tryptophan Metabolism	60	4.16	5	0.404	1	1	
Purine Metabolism	74	5.13	6	0.408	1	1	
Glutathione Metabolism	21	1.46	2	0.434	1	1	
Aspartate Metabolism	35	2.43	3	0.443	1	1	
Glutamate Metabolism	49	3.4	4	0.446	1	1	
Transfer of Acetyl Groups into Mitochondria	22	1.53	2	0.459	1	1	
Phenylacetate Metabolism	9	0.624	1	0.478	1	1	
Lactose Degradation	9	0.624	1	0.478	1	1	
Nicotinate and Nicotinamide Metabolism	37	2.57	3	0.48	1	1	
Malate-Aspartate Shuttle	10	0.693	1	0.514	1	1	
Pyruvaldehyde Degradation	10	0.693	1	0.514	1	1	
Glycolysis	25	1.73	2	0.528	1	1	
Sphingolipid Metabolism	40	2.77	3	0.534	1	1	
Cysteine Metabolism	26	1.8	2	0.549	1	1	
Warburg Effect	58	4.02	4	0.583	1	1	
Histidine Metabolism	43	2.98	3	0.585	1	1	
Lysine Degradation	30	2.08	2	0.629	1	1	
Beta-Alanine Metabolism	34	2.36	2	0.698	1	1	
Beta Oxidation of Very Long Chain Fatty Acids	17	1.18	1	0.708	1	1	
Gluconeogenesis	35	2.43	2	0.713	1	1	
Arginine and Proline Metabolism	53	3.67	3	0.728	1	1	
Galactose Metabolism	38	2.63	2	0.756	1	1	
Lactose Synthesis	20	1.39	1	0.766	1	1	
Glycine and Serine Metabolism	59	4.09	3	0.794	1	1	
Carnitine Synthesis	22	1.53	1	0.798	1	1	
Valine, Leucine and Isoleucine Degradation	60	4.16	3	0.803	1	1	
Propanoate Metabolism	42	2.91	2	0.804	1	1	
Oxidation of Branched Chain Fatty Acids	26	1.8	1	0.849	1	1	
Phenylalanine and Tyrosine Metabolism	28	1.94	1	0.87	1	1	
Selenoamino Acid Metabolism	28	1.94	1	0.87	1	1	
Arachidonic Acid Metabolism	69	4.78	3	0.874	1	1	
Pentose Phosphate Pathway	29	2.01	1	0.879	1	1	
Folate Metabolism	29	2.01	1	0.879	1	1	
Starch and Sucrose Metabolism	31	2.15	1	0.896	1	1	
Citric Acid Cycle	32	2.22	1	0.903	1	1	
Fructose and Mannose Degradation	32	2.22	1	0.903	1	1	
Methionine Metabolism	43	2.98	1	0.957	1	1	
Pyruvate Metabolism	48	3.33	1	0.971	1	1	
Tyrosine Metabolism	72	4.99	1	0.995	1	1	

Supplemental Table 2b. Results of Metabolite Set Enrichment Analyses (MSEA) for the VDD-CC011 signature.

**Metaboanalyst Pathway-SMHDP-Run 2/14/20**	**total**	**expected**	**hits**	**Raw p**	**Holm p**	**FDR**	**Metabolites in significantly enriched pathways**
Alpha Linolenic Acid and Linoleic Acid Metabolism	19	1.13	4	**0.0225**	1	1	Docosapentaenoic acid (22n-6); Docosahexaenoic acid; Adrenic acid; 8,11,14-Eicosatrienoic acid
Urea Cycle	29	1.73	5	**0.0249**	1	1	L-Alanine; L-Aspartic acid; Ornithine; Urea; L-Glutamine
Ammonia Recycling	32	1.91	5	**0.0368**	1	1	Glycine; L-Histidine; L-Serine; L-Aspartic acid; L-Glutamine
Glycine and Serine Metabolism	59	3.51	6	0.132	1	1	
Arginine and Proline Metabolism	53	3.16	5	0.203	1	1	
Methylhistidine Metabolism	4	0.238	1	0.218	1	1	
Methionine Metabolism	43	2.56	4	0.25	1	1	
Alanine Metabolism	17	1.01	2	0.269	1	1	
Pyrimidine Metabolism	59	3.51	5	0.271	1	1	
Spermidine and Spermine Biosynthesis	18	1.07	2	0.292	1	1	
Beta-Alanine Metabolism	34	2.03	3	0.33	1	1	
Glutamate Metabolism	49	2.92	4	0.333	1	1	
Glutathione Metabolism	21	1.25	2	0.359	1	1	
Carnitine Synthesis	22	1.31	2	0.381	1	1	
Phenylacetate Metabolism	9	0.536	1	0.426	1	1	
Homocysteine Degradation	9	0.536	1	0.426	1	1	
Purine Metabolism	74	4.41	5	0.455	1	1	
Malate-Aspartate Shuttle	10	0.596	1	0.46	1	1	
Tryptophan Metabolism	60	3.57	4	0.486	1	1	
D-Arginine and D-Ornithine Metabolism	11	0.655	1	0.493	1	1	
Selenoamino Acid Metabolism	28	1.67	2	0.506	1	1	
Phosphatidylethanolamine Biosynthesis	12	0.715	1	0.523	1	1	
Glucose-Alanine Cycle	13	0.774	1	0.552	1	1	
Aspartate Metabolism	35	2.08	2	0.63	1	1	
Nicotinate and Nicotinamide Metabolism	37	2.2	2	0.661	1	1	
Valine, Leucine and Isoleucine Degradation	60	3.57	3	0.71	1	1	
Lactose Synthesis	20	1.19	1	0.711	1	1	
Threonine and 2-Oxobutanoate Degradation	20	1.19	1	0.711	1	1	
Pantothenate and CoA Biosynthesis	21	1.25	1	0.728	1	1	
Betaine Metabolism	21	1.25	1	0.728	1	1	
Histidine Metabolism	43	2.56	2	0.741	1	1	
Arachidonic Acid Metabolism	69	4.11	3	0.797	1	1	
Phenylalanine and Tyrosine Metabolism	28	1.67	1	0.825	1	1	
Amino Sugar Metabolism	33	1.97	1	0.873	1	1	
Porphyrin Metabolism	40	2.38	1	0.918	1	1	
Sphingolipid Metabolism	40	2.38	1	0.918	1	1	
Propanoate Metabolism	42	2.5	1	0.928	1	1	
Warburg Effect	58	3.46	1	0.974	1	1	
Bile Acid Biosynthesis	65	3.87	1	0.984	1	1	
Tyrosine Metabolism	72	4.29	1	0.99	1	1	

Supplemental Table 3b. Results of Metabolite Set Enrichment Analyses (MSEA) for the VDS-CC042 signature.

Metaboanalyst Pathway-SMHDP-Run 2/14/20	total	expected	hits	Raw p	Holm p	FDR	Metabolites in significantly enriched pathways
Arginine and Proline Metabolism	53	2.9	6	0.0633	1	1	
Spermidine and Spermine Biosynthesis	18	0.984	3	0.0705	1	1	
Pantothenate and CoA Biosynthesis	21	1.15	3	0.102	1	1	
Methylhistidine Metabolism	4	0.219	1	0.202	1	1	
Urea Cycle	29	1.59	3	0.208	1	1	
Citric Acid Cycle	32	1.75	3	0.253	1	1	
Beta-Alanine Metabolism	34	1.86	3	0.283	1	1	
Aspartate Metabolism	35	1.91	3	0.299	1	1	
Catecholamine Biosynthesis	20	1.09	2	0.3	1	1	
Carnitine Synthesis	22	1.2	2	0.341	1	1	
Transfer of Acetyl Groups into Mitochondria	22	1.2	2	0.341	1	1	
Purine Metabolism	74	4.05	5	0.38	1	1	
Estrone Metabolism	24	1.31	2	0.382	1	1	
Phenylacetate Metabolism	9	0.492	1	0.398	1	1	
De Novo Triacylglycerol Biosynthesis	9	0.492	1	0.398	1	1	
Glycine and Serine Metabolism	59	3.23	4	0.406	1	1	
Methionine Metabolism	43	2.35	3	0.422	1	1	
Malate-Aspartate Shuttle	10	0.547	1	0.432	1	1	
Phenylalanine and Tyrosine Metabolism	28	1.53	2	0.46	1	1	
Cardiolipin Biosynthesis	11	0.602	1	0.463	1	1	
Pentose Phosphate Pathway	29	1.59	2	0.478	1	1	
Glutamate Metabolism	49	2.68	3	0.51	1	1	
Ketone Body Metabolism	13	0.711	1	0.521	1	1	
Thyroid hormone synthesis	13	0.711	1	0.521	1	1	
Ammonia Recycling	32	1.75	2	0.532	1	1	
Phosphatidylcholine Biosynthesis	14	0.766	1	0.547	1	1	
Amino Sugar Metabolism	33	1.8	2	0.549	1	1	
Tyrosine Metabolism	72	3.94	4	0.566	1	1	
Nicotinate and Nicotinamide Metabolism	37	2.02	2	0.613	1	1	
Beta Oxidation of Very Long Chain Fatty Acids	17	0.93	1	0.619	1	1	
Alanine Metabolism	17	0.93	1	0.619	1	1	
Pyrimidine Metabolism	59	3.23	3	0.64	1	1	
Sphingolipid Metabolism	40	2.19	2	0.656	1	1	
Butyrate Metabolism	19	1.04	1	0.66	1	1	
Ethanol Degradation	19	1.04	1	0.66	1	1	
Ubiquinone Biosynthesis	20	1.09	1	0.679	1	1	
Lactose Synthesis	20	1.09	1	0.679	1	1	
Threonine and 2-Oxobutanoate Degradation	20	1.09	1	0.679	1	1	
Glutathione Metabolism	21	1.15	1	0.697	1	1	
Betaine Metabolism	21	1.15	1	0.697	1	1	
Sulfate/Sulfite Metabolism	22	1.2	1	0.714	1	1	
Caffeine Metabolism	24	1.31	1	0.745	1	1	
Glycerolipid Metabolism	25	1.37	1	0.759	1	1	
Oxidation of Branched Chain Fatty Acids	26	1.42	1	0.773	1	1	
Phytanic Acid Peroxisomal Oxidation	26	1.42	1	0.773	1	1	
Plasmalogen Synthesis	26	1.42	1	0.773	1	1	
Mitochondrial Beta-Oxidation of Short Chain Saturated Fatty Acids	27	1.48	1	0.785	1	1	
Mitochondrial Beta-Oxidation of Medium Chain Saturated Fatty Acids	27	1.48	1	0.785	1	1	
Mitochondrial Beta-Oxidation of Long Chain Saturated Fatty Acids	28	1.53	1	0.797	1	1	
Phospholipid Biosynthesis	29	1.59	1	0.809	1	1	
Lysine Degradation	30	1.64	1	0.82	1	1	
Warburg Effect	58	3.17	2	0.841	1	1	
Androgen and Estrogen Metabolism	33	1.8	1	0.848	1	1	
Tryptophan Metabolism	60	3.28	2	0.855	1	1	
Fatty Acid Elongation In Mitochondria	35	1.91	1	0.865	1	1	
Fatty Acid Biosynthesis	35	1.91	1	0.865	1	1	
Retinol Metabolism	37	2.02	1	0.88	1	1	
Bile Acid Biosynthesis	65	3.55	2	0.885	1	1	
Porphyrin Metabolism	40	2.19	1	0.899	1	1	
Propanoate Metabolism	42	2.3	1	0.91	1	1	
Histidine Metabolism	43	2.35	1	0.915	1	1	
Fatty acid Metabolism	43	2.35	1	0.915	1	1	
Steroid Biosynthesis	48	2.62	1	0.937	1	1	
Pyruvate Metabolism	48	2.62	1	0.937	1	1	
Valine, Leucine and Isoleucine Degradation	60	3.28	1	0.969	1	1	

Supplemental Table 1b. Results of Metabolite Set Enrichment Analyses (MSEA) for the VDD-CC017 signature.

**Metaboanalyst Pathway-SMHDP-Run 2/14/20**	**total**	**expected**	**hits**	**Raw p**	**Holm p**	**FDR**	**Metabolites in significantly enriched pathways**
Pantothenate and CoA Biosynthesis	21	1.19	5	**0.00495**	0.485	0.485	Cytidine monophosphate; L-Cysteine; Dephospho-CoA; Pantetheine 4'-phosphate; Coenzyme A
Glutathione Metabolism	21	1.19	4	**0.0269**	1	1	Cysteinylglycine; Glutathione; L-Cysteine; gamma-Glutamylcysteine
Homocysteine Degradation	9	0.51	2	0.0878	1	1	
Cardiolipin Biosynthesis	11	0.623	2	0.125	1	1	
Taurine and Hypotaurine Metabolism	12	0.68	2	0.145	1	1	
Glutamate Metabolism	49	2.78	4	0.299	1	1	
Phenylacetate Metabolism	9	0.51	1	0.41	1	1	
De Novo Triacylglycerol Biosynthesis	9	0.51	1	0.41	1	1	
Glycerolipid Metabolism	25	1.42	2	0.419	1	1	
Glycine and Serine Metabolism	59	3.34	4	0.433	1	1	
Cysteine Metabolism	26	1.47	2	0.439	1	1	
Plasmalogen Synthesis	26	1.47	2	0.439	1	1	
Pyruvaldehyde Degradation	10	0.566	1	0.443	1	1	
Methionine Metabolism	43	2.44	3	0.445	1	1	
Mitochondrial Beta-Oxidation of Long Chain Saturated Fatty Acids	28	1.59	2	0.479	1	1	
Phosphatidylethanolamine Biosynthesis	12	0.68	1	0.505	1	1	
Ketone Body Metabolism	13	0.736	1	0.534	1	1	
Vitamin K Metabolism	14	0.793	1	0.56	1	1	
Phosphatidylcholine Biosynthesis	14	0.793	1	0.56	1	1	
Amino Sugar Metabolism	33	1.87	2	0.569	1	1	
Aspartate Metabolism	35	1.98	2	0.602	1	1	
Beta Oxidation of Very Long Chain Fatty Acids	17	0.963	1	0.632	1	1	
Phosphatidylinositol Phosphate Metabolism	17	0.963	1	0.632	1	1	
Nicotinate and Nicotinamide Metabolism	37	2.1	2	0.633	1	1	
Retinol Metabolism	37	2.1	2	0.633	1	1	
Galactose Metabolism	38	2.15	2	0.647	1	1	
Spermidine and Spermine Biosynthesis	18	1.02	1	0.653	1	1	
Butyrate Metabolism	19	1.08	1	0.673	1	1	
Ethanol Degradation	19	1.08	1	0.673	1	1	
Threonine and 2-Oxobutanoate Degradation	20	1.13	1	0.692	1	1	
Propanoate Metabolism	42	2.38	2	0.702	1	1	
Transfer of Acetyl Groups into Mitochondria	22	1.25	1	0.727	1	1	
Caffeine Metabolism	24	1.36	1	0.757	1	1	
Pyruvate Metabolism	48	2.72	2	0.771	1	1	
Oxidation of Branched Chain Fatty Acids	26	1.47	1	0.785	1	1	
Phytanic Acid Peroxisomal Oxidation	26	1.47	1	0.785	1	1	
Inositol Phosphate Metabolism	26	1.47	1	0.785	1	1	
Mitochondrial Beta-Oxidation of Short Chain Saturated Fatty Acids	27	1.53	1	0.797	1	1	
Mitochondrial Beta-Oxidation of Medium Chain Saturated Fatty Acids	27	1.53	1	0.797	1	1	
Purine Metabolism	74	4.19	3	0.808	1	1	
Phospholipid Biosynthesis	29	1.64	1	0.82	1	1	
Pentose Phosphate Pathway	29	1.64	1	0.82	1	1	
Urea Cycle	29	1.64	1	0.82	1	1	
Lysine Degradation	30	1.7	1	0.831	1	1	
Starch and Sucrose Metabolism	31	1.76	1	0.84	1	1	
Citric Acid Cycle	32	1.81	1	0.85	1	1	
Fructose and Mannose Degradation	32	1.81	1	0.85	1	1	
Inositol Metabolism	33	1.87	1	0.859	1	1	
Beta-Alanine Metabolism	34	1.93	1	0.867	1	1	
Valine, Leucine and Isoleucine Degradation	60	3.4	2	0.869	1	1	
Fatty Acid Elongation In Mitochondria	35	1.98	1	0.875	1	1	
Fatty Acid Biosynthesis	35	1.98	1	0.875	1	1	
Histidine Metabolism	43	2.44	1	0.923	1	1	
Fatty acid Metabolism	43	2.44	1	0.923	1	1	
Steroid Biosynthesis	48	2.72	1	0.943	1	1	
Arginine and Proline Metabolism	53	3	1	0.958	1	1	
Warburg Effect	58	3.29	1	0.969	1	1	
Pyrimidine Metabolism	59	3.34	1	0.971	1	1	
Tryptophan Metabolism	60	3.4	1	0.973	1	1	
Bile Acid Biosynthesis	65	3.68	1	0.98	1	1	
Arachidonic Acid Metabolism	69	3.91	1	0.985	1	1	

Supplemental Table 5b. Results of Metabolite Set Enrichment Analyses (MSEA) for the VDD signature after adjustment for strain.

**Metaboanalyst Pathway-"previous KEGG"-Run 8/9/19**	**total**	**expected**	**hits**	**Raw p value**	**FDR**	**Metabolites in significantly enriched pathways**
Biosynthesis of unsaturated fatty acids	42	1.0967	7	**0.0001**	**0.0052**	Icosenoic acid; Icosadienoic acid; Palmitic acid; Stearic acid; Oleic acid; Linoleic acid; Eicosapentaenoic acid
Glycerophospholipid metabolism	30	0.78335	5	**0.0008**	**0.0334**	Phosphatidylethanolamine; Phosphatidylcholine; LysoPC(18:1(9Z)); Glycerylphosphorylethanolamine; Glycerophosphocholine
Linoleic acid metabolism	6	0.15667	2	**0.0093**	0.2550	Linoleic acid; Phosphatidylcholine
Fatty acid biosynthesis	43	1.1228	4	**0.0234**	0.4150	Myristic acid; Stearic acid; Oleic acid; Palmitic acid
Arginine and proline metabolism	44	1.1489	4	**0.0253**	0.4150	Argininosuccinic acid; L-Arginine; Putrescine;S-Adenosylmethionine
Sulfur metabolism	5	0.13056	1	0.1240	1.0000	
Cysteine and methionine metabolism	27	0.70501	2	0.1550	1.0000	
alpha-Linolenic acid metabolism	9	0.235	1	0.2120	1.0000	
Purine metabolism	68	1.7756	3	0.2600	1.0000	
Nicotinate and nicotinamide metabolism	13	0.33945	1	0.2920	1.0000	
Glycosylphosphatidylinositol(GPI)-anchor biosynthesis	14	0.36556	1	0.3110	1.0000	
Sphingolipid metabolism	21	0.54834	1	0.4290	1.0000	
Lysine degradation	23	0.60056	1	0.4580	1.0000	
Alanine, aspartate and glutamate metabolism	24	0.62668	1	0.4730	1.0000	
Glutathione metabolism	26	0.6789	1	0.5010	1.0000	
Fatty acid elongation in mitochondria	27	0.70501	1	0.5140	1.0000	
Arachidonic acid metabolism	36	0.94001	1	0.6190	1.0000	
Fatty acid metabolism	39	1.0183	1	0.6490	1.0000	
Pyrimidine metabolism	41	1.0706	1	0.6670	1.0000	
Aminoacyl-tRNA biosynthesis	69	1.8017	1	0.8460	1.0000	
Steroid hormone biosynthesis	72	1.88	1	0.8580	1.0000	
